# Supplementary material for: The utility of diagnostic selective nerve root blocks in the management of patients with lumbar radiculopathy: a systematic review
Source: BMJ Open. 2019 Apr 20;9(4):e025790. doi: 10.1136/bmjopen-2018-025790 (PMC6500311; doi:10.1136/bmjopen-2018-025790)
Supplement: Supplementary data [file bmjopen-2018-025790supp001.pdf]

ELECTRONIC DATABASE SEARCH STRATEGY:
Spinal nerve block- diagnostic utility in back pain

Database: Ovid MEDLINE(R) Epub Ahead of Print, In-Process & Other Non-Indexed Citations, Ovid MEDLINE(R) Daily and Ovid MEDLINE(R) <1946 to Present>

Search Strategy:

----

1 exp Back Pain/ (34777)
2 back pain.tw. (39654)
3 backache.tw. (2333)
4 Radiculopathy/ (4527)
5 Lumbar Vertebrae/ (46628)
6 Lumbosacral Region/ (11245)
7 radiculopath$.tw. (5409)
8 lumbago.tw. (1264)
9 lumber.tw. (1065)
10 lumbosacral.tw. (10151)
11 radiculitis.tw. (781)
12 (radicular adj3 pain).tw. (2652)
13 spinal pain.tw. (1247)
14 exp Spinal Nerve Roots/ (29515)
15 Sciatica/ (4816)
16 lumbar.tw. (93988)
17 sciatica.tw. (3914)
18 Intervertebral Disk Displacement/ (17468)
19 Zygapophyseal Joint/ (1525)
20 Spinal Stenosis/ (5278)
21 Foraminal Stenosis.tw. (515)
22 Foramenal Stenosis.tw. (3)
23 lateral recess stenosis.tw. (124)
24 or/1-23 (195289)
25 exp Nerve Block/ (19509)
26 (nerve adj3 block$).tw. (11732)
27 SNRB.tw. (39)
28 (transforaminal adj3 injection$).tw. (523)
29 Injections, Epidural/ (2600)
30 (neural adj3 block$).tw. (979)
31 (nerve adj3 injection$).tw. (1375)
32 (nerve adj3 infiltration).tw. (583)
33 (block adj3 an?esthetic$).tw. (1128)

34 exp Injections, Spinal/ (15080)

35 facet block$.tw. (80)

36 facet injection$.tw. (69)

37 epidural injection$.tw. (1674)

38 Injections, Intra-Articular/ (6865)

39 diagnostic injection$.tw. (139)

40 or/25-39 (49688)

41 24 and 40 (6586)

42 exp Anesthetics, Local/ (99162)

43 lidocaine.tw. (19794)

44 lignocaine.tw. (2740)

45 local an?esthetic$.tw. (22917)

46 bupivacaine.tw. (11894)

47 exp Triamcinolone/ (8898)

48 Triamcinolone.tw. (6896)

49 volon.tw. (33)

50 aristocort.tw. (22)

51 Depo-medrone.tw. (16)

52 Depomedrone.tw. (20)

53 Steroids/ (34837)

54 Depo steroid$.tw. (7)

55 Deposteroid$.tw. (8)

56 kenalog.tw. (195)

57 kenacort.tw. (59)

58 Depo-Medrol.tw. (146)

59 depomedrol.tw. (40)

60 exp Betamethasone/ (6913)

61 betamethasone.tw. (4443)

62 exp prednisolone/ (48285)

63 prednisolone.tw. (23608)

64 methylprednisolone.tw. (14255)

65 ((steroid$ or corticosteroid$) adj5 (injection$ or infiltration or block)).tw. (8468)

66 or/42-65 (233944)

67 Diagnosis/ (17032)

68 diagnosis, differential/ (424334)

69 diagnosis.fs. (2333150)

70 diagnos$.tw. (2126562)

71 or/67-70 (3705304)

72 66 and 71 (36366)

73 24 and 72 (1633)

74 41 or 73 (7546)

75 exp animals/ not humans/ (4436130)

76 74 not 75 (6125)

77 (20101* or 2011* or 2012* or 2013* or 2014* or 2015* or 2016* or 2017* or 2018*).ep,ez,dc,dp. (7686055)

78 76 and 77 (2085)

Proquest Dissertations and Theses Global

all(backache OR lumbar OR "back pain" OR radiculopathy) AND all("transforaminal injection" OR "back pain infiltration" OR "back pain block" OR "facet injection" OR SNRB OR "nerve block" OR "nerve root block" OR "nerve infiltration" OR "selective nerve root infiltration" OR "facet block" OR "radiculopathy block" OR "radiculopathy infiltration")
