# Supplementary material for: The utility of diagnostic selective nerve root blocks in the management of patients with lumbar radiculopathy: a systematic review
Source: BMJ Open. 2019 Apr 20;9(4):e025790. doi: 10.1136/bmjopen-2018-025790 (PMC6500311; doi:10.1136/bmjopen-2018-025790)
Supplement: Supplementary data [file bmjopen-2018-025790supp002.pdf]

Supplementary Table 1 Details of included studies

Author (Year) Country	N analysed/N recruited	Inclusion criteria	Description of included patients	Details of previous surgery	Needle level	Anaesthetic details	Guided method	Needle provocation	Number of control injections	Time to pain measurement
Within patient case-control studies										
Yeom[26] (2008) NR	47/83	Patients due to undergo a lumbar spine operation with single-level, unilateral lumbosacral radiculopathy confirmed by clinical, radiographic & MRI findings.	Patients with established pure radiculopathy from a single level. Affected roots were L4 in 3, L5 in 31, S1 in 13. Concordant imaging & clinical findings.	No history of lumbar surgeries	L3, L4, L5, S1	1ml of 2% Lidocaine	Fluoroscopy	No	1 or 2	30 minutes
North[29] (1996) USA	33/33	Patients with sciatica with or without low back pain, attributed to spinal pathology.	Established sciatica patients with or without low back pain. All had L5 or S1 radiculopathy. 52% had diagnostic imaging findings of ongoing nerve root compression. The remaining 48% had a well-documented history of root compression which had been corrected surgically.	48% history of root compression corrected surgically.	L5, S1	3ml of 0.5% Bupivacaine	Fluoroscopy	Yes	3	Every 15 minutes for 3 hours
Prospective diagnostic cohort studies										
Schutz[31] (1973) Canada	15/23	Patients with current sciatica.	Patients with sciatica. Investigation undertaken only at a time when sciatica symptoms actually present.	1 patient had previous surgery, unsuccessful SNRB & excluded from analysis. Unclear if patients	NR	1ml of Procaine (concentrati	Guided but method not reported	Yes	1 or 2	Immediate

Author (Year) Country	N analysed/N recruited	Inclusion criteria	Description of included patients	Details of previous surgery	Needle level	Anaesthetic details	Guided method	Needle provocation	Number of control injections	Time to pain measurement
				included in analysis had previous surgeries.		on not reported)				
Retrospective diagnostic cohort studies										
Sasso[29] (2005) USA	83/83	Patients who underwent SNRB, MRI & nerve root decompression surgery and had a follow-up evaluation >12 months post surgery	Patients with cervical or lumbar radiculopathy. Discordant imaging and clinical findings	Unclear how many previous lumbar surgeries. 20 patients with cervical or lumbar symptoms had previous surgery	NR	0.5-0.7ml of 2% Lidocaine	Fluoroscopy	Yes	NR	Immediate
Dooley[32] (1988) Canada	62/73	Patients who underwent nerve root infiltration	Patients with radicular pain who underwent nerve root infiltration	32 >=1 previous surgery, 3 had 4 surgeries.	L3, L4, L5, S1	1ml of 1% Mepivacaine or Lidocaine	Fluoroscopy	Yes	NR	Immediate
Williams[28] (2015) UK	96/100	Patients who underwent diagnostic lumbar DRGB (identified retrospectively)	Patients with presumed radicular leg pain with significant diagnostic uncertainty from the patient's presenting history, examination and imaging as to whether lumbosacral nerve root compression was indeed responsible.	NR	L1, L3, L4, L5, S1	2 mL of 1% Lidocaine and 0.5 to 1 mL of Iopamidol	Fluoroscopy	Yes	NR	Immediate

Abbreviations: DRGB, dorsal root ganglion block; NR, not reported; MRI, Magnetic resonance imaging; SNRB, selective nerve root block.
