# Supplementary material for: The utility of diagnostic selective nerve root blocks in the management of patients with lumbar radiculopathy: a systematic review
Source: BMJ Open. 2019 Apr 20;9(4):e025790. doi: 10.1136/bmjopen-2018-025790 (PMC6500311; doi:10.1136/bmjopen-2018-025790)
Supplement: Supplementary data [file bmjopen-2018-025790supp003.pdf]

Supplementary Table 2 Patient characteristics of studies reporting on adverse events following SNRB

Author (year) Country	N analysed/ N recruited	Inclusion criteria	Needle tip position	Needle levels	Anaesthetic details	Guided method	Needle provocation used?	Adverse events
Case-series								
Stalcup[32] (2006) USA	1203/ 1203	All adult patients who underwent a SNRB in the lumbar spine in a radiology department.	Adjacent or into the intervertebral foramen.	NR	1-2ml of 0.25% Bupivacaine	Fluoroscopy	NR	Numbers given in injections: Leg weakness n=77; Pain increased n=51; Other n=6; Total n=134
Ng[35] (2004) UK	117/ 125	Consecutive patients with clinical evidence of unilateral radicular pain that lasted despite at least 6 weeks of conservative management, MRI confirmation of nerve root compression secondary to lumbar disc herniation or peripheral degenerative spinal stenosis.	Superiorly to pedicle, medially to nerve and laterally to vertebral body.	NR	2ml of 0.25% Bupivacaine	Assumed Fluoroscopy	NR	No adverse events
Jonsson[34] (1988) Sweden	78/ 78	Patients with unilateral sciatic pain, considered for surgery. Sciatic pain but normal findings on myelography, CT and/or MRI.	Just lateral to the opening of the intervertebral foramen.	L4, L5, S1	3-6ml of Carbocaine (% NR)	Fluoroscopy	NR	No adverse events.
Quinn[36] (1988) USA	33/ 33	Patients with a herniated disc or foraminal stenosis (n=2) as identified by CT or MRI.	An attempt was made to pierce the nerve or to have the needle tip within 1-2mm of the nerve.	NR	2.5-5ml of 1% Lidocaine or 0.5% Bupivacaine	CT	Yes	No adverse events.
Tajima[37] (1980) Japan	106/ 106	Patients with radicular symptoms undergoing lumbosacral radiculography and block who had lumbosacral diseases.	Approx 4cm lateral to upper margin of lumbar spinous process corresponding to nerve root to be radiographed.	L4, L5, S1	3ml of 1% Lidocaine	x-ray	Yes	Pain in the lower extremity was aggravated for 1-2 days

								following selective radiculography and block in 4 patients. There was no other complication.
Diagnostic cohort study								
*Schutz[31] (1973) Canada	15/ 23	Patients with current sciatica.	Superior level of intervertebral foramen. Introduced about 2" from the midline.	NR	1ml Procaine	Guided but method not reported	Yes	No adverse events.
*Williams[28] (2015) UK	96/100	Patients with presumed radicular leg pain who underwent diagnostic lumbar DRGB (identified retrospectively).	Inserted from a paraspinal entry point and advanced to the superoanterior margin of the intervertebral foramen of the targeted level.	L1,L3, L4,L5 & S1	2 mL of 1% Lidocaine and 0.5 to 1 mL of Iopamidol	Fluoroscopy	Yes	No adverse events.
Randomized controlled trial								
Ghahreman[33] (2010) Australia	27/ 150	Adult patients with lower limb radiculopathy; limitation of straight-leg raise to <30º; disc herniation on CT or MRI. Considered for surgery. Only data for single arm of trial in which patients received anaesthetic was included in the current review.	Placed in the intervertebral foramen of the target level.	L2,L3, L4,L5 & S1	2ml of 0.5% Bupivacaine	Assumed Fluoroscopy	NR	No complications occurred that could be attributed to the treatment.

*Included in diagnostic accuracy systematic review

Abbreviations: CT, computerised tomography; DRGB, dorsal root ganglion block; MRI, magnetic resonance imaging; NR, not reported; SNRB, selective nerve root block.
