# Supplementary material for: The utility of diagnostic selective nerve root blocks in the management of patients with lumbar radiculopathy: a systematic review
Source: BMJ Open. 2019 Apr 20;9(4):e025790. doi: 10.1136/bmjopen-2018-025790 (PMC6500311; doi:10.1136/bmjopen-2018-025790)
Supplement: Author's manuscript [file bmjopen-2018-025790.draft_revisions.pdf]

BMJ Open

BMJ Open is committed to open peer review. As part of this commitment we make the peer review history of every article we publish publicly available.

When an article is published we post the peer reviewers' comments and the authors' responses online. We also post the versions of the paper that were used during peer review. These are the versions that the peer review comments apply to.

The versions of the paper that follow are the versions that were submitted during the peer review process. They are not the versions of record or the final published versions. They should not be cited or distributed as the published version of this manuscript.

BMJ Open is an open access journal and the full, final, typeset and author-corrected version of record of the manuscript is available on our site with no access controls, subscription charges or pay-per-view fees (http://bmjopen.bmj.com).

If you have any questions on BMJ Open's open peer review process please email info.bmjopen@bmj.com

BMJ Open

The utility of diagnostic selective nerve root blocks in the management of patients with lumbar radiculopathy: a systematic review

Journal:	*BMJ Open*
Manuscript ID	bmjopen-2018-025790
Article Type:	Research
Date Submitted by the Author:	06-Aug-2018
Complete List of Authors:	Beynon, Rebecca; University of Bristol, Population Health Sciences, Bristol Medical School<br>Elwenspoek, Martha; University Hospitals Bristol NHS Foundation Trust, The National Institute for Health Research Collaboration for Leadership in Applied Health Research and Care West (NIHR CLAHRC West); University of Bristol, Population Health Sciences, Bristol Medical School<br>Sheppard, Athena; University of Bristol, Population Health Sciences, Bristol Medical School; University Hospitals Bristol NHS Foundation Trust, The National Institute for Health Research Collaboration for Leadership in Applied Health Research and Care West (NIHR CLAHRC West)<br>Kolias, Angelos; Addenbrooke’s Hospital & University of Cambridge, Division of Neurosurgery, Department of Clinical Neurosciences<br>Laing, Rodney; Addenbrooke’s Hospital & University of Cambridge, Division of Neurosurgery, Department of Clinical Neurosciences<br>Whiting, Penny; University of Bristol, Population Health Sciences, Bristol Medical School; University Hospitals Bristol NHS Foundation Trust, The National Institute for Health Research Collaboration for Leadership in Applied Health Research and Care West (NIHR CLAHRC West)<br>Hollingworth, William; University of Bristol, Population Health Sciences, Bristol Medical School
Keywords:	diagnostic accuracy, selective nerve root blocks, lumbar radiculopathy, low back pain, lumbar decompression surgery

SCHOLARONE™
Manuscripts

The utility of diagnostic selective nerve root blocks in the management of patients with lumbar radiculopathy: a systematic review

Rebecca Beynon[2], Martha MC Elwenspoek[1,2], Athena Sheppard[1,2], Angelos G Kolias[3], Rodney J Laing[3], Penny Whiting[1,2], William Hollingworth[2]

Affiliations:

1. The National Institute for Health Research Collaboration for Leadership in Applied Health Research and Care West (NIHR CLAHRC West), University Hospitals Bristol NHS Foundation Trust, Bristol, UK
2. Population Health Sciences, Bristol Medical School, University of Bristol, Bristol, UK
3. Division of Neurosurgery, Department of Clinical Neurosciences, Addenbrooke's Hospital & University of Cambridge, Cambridge, UK

Corresponding author:

Martha M C Elwenspoek

9th Floor, Whitefriars, Lewins Mead,

Bristol, BS1 2NT

Email: martha.elwenspoek@bristol.ac.uk

Tel: +44/0 117 3427275

Word count: 3110

For peer review only

ABSTRACT

Objective: Lumbar radiculopathy (LR) causes low back pain accompanied by pain radiating to the legs. Unsuccessful back surgery is associated with significant healthcare costs and risks to patients. This review aims to examine the diagnostic accuracy of selective nerve root blocks (SNRB) to identify patients most likely to benefit from lumbar decompression surgery.

Design: Systematic review of diagnostic test accuracy studies.

Eligibility criteria: Primary research articles using a patient population with low back pain and symptoms in a lower limb, SNRB administered under radiological guidance as index test, and any reported reference standard for the diagnosis of LR.

Information sources: MEDLINE (Ovid), MEDLINE In-Process & Other Non-Indexed Citations, EMBASE, Science Citation Index, Biosis and LILACS, Dissertation abstracts and NTIS from inception to 2018.

Methods: Risk of bias and applicability was assessed using the QUADAS-2 tool. We performed random effects logistic regression to meta-analyse studies grouped by reference standard.

Results: 6 studies (341 patients) were included in this review. All studies were judged at high risk of bias. There was substantial heterogeneity across studies in sensitivity (range 57-100%) and specificity (10-86%) estimates. Four studies were diagnostic cohort studies that used either intra-operative findings during surgery (pooled sensitivity: 93.5% [95% CI 84.0-97.6]; specificity: 50.0% [16.8-83.2]) or 'outcome following surgery' as the reference standard (pooled sensitivity: 90.9% [83.1-95.3]; specificity 22.0% [7.4- 49.9]). Two studies had a within-patient case-control study design, but results were not pooled because different types of control injections were used.

Conclusions: We found limited evidence which was of low methodological quality indicating that the diagnostic accuracy of SNRB is uncertain and that specificity in particular may be low. SNRB is a safe test with a low risk of clinically significant complications, but it remains unclear whether the additional diagnostic information it provides justifies the cost of the test.

Keywords: diagnostic accuracy of selective nerve root blocks (SNRB), lumbar radiculopathy, low back pain, lumbar decompression surgery.

For peer review only - http://bmjopen.bmj.com/site/about/guidelines.xhtml

ARTICLE SUMMARY

Strengths and limitations of this study

- Comprehensive synthesis of the current evidence on diagnostic accuracy of SNRB in lumbar radiculopathy.
- Extensive literature searches were conducted using several databases without restrictions on publication date, language, or study type, in an attempt to locate all relevant studies.
- We used rigorous eligibility criteria, which excluded studies with mixed cervical and lumbar spine pathology and studies where there was insufficient data to construct estimates of sensitivity and specificity.
- Only a small number of primary diagnostic accuracy studies could be included in our review and all had methodological limitations.
- Due to the small number of studies, we were unable to explore the value of SNRB in potentially important patient subgroups, such as those with suspected multi-level radiculopathy.

For peer review only

For peer review only - http://bmjopen.bmj.com/site/about/guidelines.xhtml

INTRODUCTION

In Western Europe, low back pain is the leading cause of disability and represents a high economic burden,[1] in particular due to production losses and cost of informal care.[1] In a subgroup of patients, low back pain is accompanied by pain radiating to the legs (radicular pain), caused by lumbar radiculopathy (LR). LR can be the result of compressive or inflammatory disorders of the spinal nerve roots or a combination of these. Randomized trial evidence on the effectiveness of lumbar decompressive surgery in patients with radiculopathy and intervertebral disc herniation suggests that early surgery leads to faster pain relief, but longer-term effectiveness is less clear.[2-7] Current UK guidelines recommend spinal decompression surgery for patients with radicular pain when non-surgical treatments have not improved symptoms and radiological findings are consistent with physical examination.[8] However, surgery does not always resolve radicular pain and 5-36% of patients suffer from recurrent back and leg pain within 2 years post-surgery.[9] The main cause of failed back surgery is inaccurate diagnosis.[10] Improved diagnosis could help identify patients most likely to benefit from surgery and minimise the cost and risks associated with unsuccessful back surgery.

A timely and accurate diagnosis of the cause of low back pain and radicular pain is important, since it is occasionally an early symptom of serious systemic disease,[11] and an inaccurate diagnosis can lead to a cascade of costly, invasive and ineffective therapy. In most patients the diagnosis of radiculopathy, caused by nerve root compression, is made by correlation of clinical signs, symptoms and imaging findings. However, neither clinical findings nor anatomical imaging have perfect diagnostic accuracy.[12] When clinical and imaging findings are equivocal or discordant uncertainty remains about the source of the symptoms and whether nerve root decompression will relieve symptoms. Additional diagnostic tests could help clinicians and patients to choose between surgical and conservative care or guide surgery in patients with suspected multi-level radiculopathy.

Diagnostic selective nerve root blocks (SNRB) inject local anaesthetic or other substances around spinal nerves under imaging guidance. Both provocative responses (replicating symptoms during needle placement) and analgesic responses (significant reduction of symptoms) to SNRB may be

For peer review only - http://bmjopen.bmj.com/site/about/guidelines.xhtml

diagnostically useful in confirming or ruling out a nerve root as the source of clinical symptoms. Some clinical guidelines and consensus statements have endorsed the use of SNRB to identify the source of pain in patients with multilevel pathology and in the pre-operative evaluation of patients with a negative or inconclusive imaging study.[13 14] Over the last decade, several systematic reviews have investigated SNRB as diagnostic tool, covering the literature up to 2012.[15-18] However, evidence was scarce and of low quality and the diagnostic accuracy and reliability of SNRB remained unclear. We updated our previous systematic review to determine the diagnostic performance of SNRB in addition to clinical and imaging findings for identifying patients with lumbar radiculopathy who are good candidates for lumbar decompression surgery.[15] A secondary aim was to summarise evidence on the incidence of procedure related complications.

For peer review only

For peer review only - http://bmjopen.bmj.com/site/about/guidelines.xhtml

MATERIALS AND METHODS

Literature search

We updated the search from our previous review, searching all databases to March 2018. Our previous search aimed to identify published and unpublished studies by searching MEDLINE (Ovid), MEDLINE In-Process & Other Non-Indexed Citations, EMBASE, Science Citation Index, Biosis and LILACS (Latin American and Caribbean literature database), Dissertation abstracts and NTIS (National Technical Information Service) from inception to March 2018. Our search strategy combined terms for SNRB with terms for sciatica or radiculopathy.[15] We did not use a methodological search filter to identify diagnostic accuracy studies as such filters result in the omission of relevant studies.[19-21] No language restrictions were applied. Attempts were made to identify further studies by examining the reference lists of all included articles.

Study selection

Studies were eligible for the diagnostic accuracy review if they included patients with low back pain with radicular pain in a lower limb who underwent SNRB under imaging guidance and reported sufficient data to construct a table detailing diagnostic accuracy (i.e. numbers of true negative, true positive, false positive, and false negative results) of the index test (SNRB) compared to any reported 'reference standard'. When we were unable to extract sufficient details from otherwise eligible studies we contacted study authors.

In diagnostic accuracy studies, the reference standard is typically a definitive test used to determine the true diagnosis, but no such definitive test exists for radicular pain due to nerve root compression. Therefore, most diagnostic studies used either intra-operative findings or post-surgical follow up as the reference standard to judge the diagnostic accuracy of SNRB. An alternative approach is to determine the sensitivity of SNRB using a 'case' injection at a symptomatic nerve root level where nerve root compression is confirmed by imaging. Specificity is evaluated by a 'control' injection at an asymptomatic site (e.g. adjacent nerve root) where imaging demonstrates no nerve root compression. Hence, in this approach concordant clinical and imaging findings are used as the reference standard.

For peer review only - http://bmjopen.bmj.com/site/about/guidelines.xhtml

Two reviewers independently screened titles and abstracts for relevance and full papers for eligibility. Any disagreements were resolved by consensus or referred to the review team.

Data extraction and quality (bias and applicability) assessment

Data extraction was performed by one reviewer and checked by a second: disagreements were resolved by consensus or discussion among co-authors. We extracted data on: study design, inclusion and exclusion criteria, included patients, SNRB details and reference standard details. 'Per patient' data were extracted: if these were unavailable we extracted 'per injection' data.

Studies included in the diagnostic review were assessed for methodological quality using the QUADAS-2 measure of bias and applicability. Bias occurs if the results of a study are distorted by flaws or limitations in its design or conduct (e.g. knowledge of the index test result when interpreting the reference standard). Applicability may be reduced if patient characteristics, or the use or interpretation of the index test in the study differ from those likely to prevail in clinical practice. Reviewers rate concerns regarding applicability and risk of bias as low, high or unclear. At least two reviewers assessed quality using QUADAS-2 and any disagreements were resolved by consensus.[22]

Studies were judged to be of high applicability if: 1) they recruited patients with low back pain and suspected radiculopathy (or sciatica) with non-congruent imaging and clinical findings who might benefit from lumbar decompression surgery; 2) the SNRB included injection of anaesthetic, sometimes in conjunction with a steroid, close to the lumbar nerve root most often under guidance by fluoroscopy or other imaging; 3) the test aimed to identify patients with radiculopathy (or sciatica) that was amenable to surgery; and 4) the reference standard was outcome of surgery. We did not carry out formal quality assessment of studies reporting on adverse events.

Data synthesis and analysis

We performed all analyses in Stata v15.1.[23] We calculated sensitivity and specificity of SNRB from each study and plotted these in receiver-operating characteristic (ROC) space. We performed random effects logistic regression to meta-analyse studies grouped by reference standard,[24] using an updated

For peer review only - http://bmjopen.bmj.com/site/about/guidelines.xhtml

version of the *metandi* package.[25] Data from studies on adverse events were combined in a narrative summary. We reported our findings according to the Preferred Reporting Items for Systematic Reviews and Meta-Analyses (PRISMA) for Diagnostic Test Accuracy (DTA) Studies.[26]

Patient and Public Involvement

Patients and members of the public were not involved in this review.

For peer review only

For peer review only - http://bmjopen.bmj.com/site/about/guidelines.xhtml

RESULTS

The original searches identified 12,883 titles and abstracts and an additional 5,267 were identified in the update search in 2018. Overall, 61 titles and abstracts were considered potentially relevant and full papers were retrieved and screened. Our original review included 5 studies. We identified one additional relevant study through our updated searches. A total of 6 studies (total 341 patients, sample size range 15 to 100) were therefore included in the review of diagnostic accuracy (Figure 1). Where reported, the mean age of patients was in the mid-forties, the majority were male, and most had had symptoms for at least 3 months. One study excluded patients with a previous history of lumbar surgery,[27] in contrast a substantial minority of patients (up to 48%) had had previous surgery in two of the other studies. Details of the patients included, and the injections delivered in each study are given in Table 1 (Supplementary Table 1).

Four diagnostic cohort studies (one prospective and three retrospective) recruited patients with suspected lumbar radiculopathy in whom some doubt remained due to equivocal or discordant clinical and radiological findings. Schutz et al. and Dooley et al. used intra-operative findings during surgery as the reference standard (Table 2).[28 29] In addition, Dooley et al. used outcome following surgery as a second reference standard.[29] Williams et al. and Sasso et al. used outcome following surgery at 3 and 12 months,[30 31] respectively, as the reference standard.

Two studies had a within-patient case-control study design. In the Yeom et al. study control injections were given at adjacent asymptomatic nerve roots,[27] whereas in the North et al. study other anatomic sites in the lumbar spine were injected (sciatic nerve, facet joint and subcutaneous).[32] All cases were confirmed by concordant clinical and radiological or surgical findings prior to the use of SNRB.

Quality of included studies

All studies were judged at high risk of bias (Table 3). All studies had high risk of bias for the reference standard because post-surgical outcomes were not considered[27 32] or selectively measured[28-31] (e.g. surgery was predominantly performed in patients with positive SNRB findings). The four cohort studies were at high risk of bias for flow and timing because patients

For peer review only - http://bmjopen.bmj.com/site/about/guidelines.xhtml

were selected to undergo surgery based on the SNRB result, with patients testing positive more likely to receive surgery. It is likely that the patients with negative SNRB results who, despite this, were selected for surgery were a biased subset of those testing negative as these are likely to have been the patients in whom the clinicians suspected a false-negative result. The two within patient case-control studies were at high risk of bias and poor applicability for patient selection because they recruited patients with unequivocal and concordant imaging and clinical findings rather than patients where diagnostic uncertainty remained. Three cohort studies were judged as low concerns regarding applicability on all domains.[29-31] There were high concerns regarding the applicability of the fourth cohort study as the reference standard consisted of intra-operative findings alone.[28]

Summary of test accuracy results

The diagnostic cohort studies reported data at the patient level, but only data at the injection level were available for the within-patient case-control studies. The threshold used to determine a positive SNRB test varied between studies (Table 2). We decided not to pool the results of studies that used different reference standards.

There was substantial heterogeneity in estimates of sensitivity and specificity across studies; sensitivity ranged from 57% to 100% and specificity from 10% to 86% (Table 2, Figure 2). Sensitivity exceeded 85% in all studies except Yeom et al. (57%).[27] Specificity was lower than 75% in all studies except Yeom et al. (86%).[27] Interpretation of specificity is particularly hampered by verification bias in the cohort studies. Because surgeons were not blinded to the SNRB results, very few patients with negative test findings had surgery. Williams et al., Sasso et al., Schutz et al., and Dooley et al. contributed a total of just ten true negative cases[28-31]. The higher specificity reported by Yeom et al. could be a manifestation of patient selection bias as 'control' injections were performed at a level of the spine where the patients had no symptoms or imaging findings suggestive of pathology.[27]

For peer review only - http://bmjopen.bmj.com/site/about/guidelines.xhtml

Due to the patient selection bias inherent in within patient case-control designs we decided that it would be inappropriate statistically to combine their results with those of the diagnostic cohort studies, and because of differences in the type of control injection we did not pool the results of the two studies. Based on the two cohort studies that used an intra-operative reference standard the pooled sensitivity was 93.5% (95% CI 84.0% - 97.6%) and specificity was 50.0% (16.8% - 83.2%). For the three studies that used post-surgery as the reference standard the summary sensitivity was 90.9% (83.1% - 95.3%) and summary specificity was 22.0% (7.4% - 49.9%). Low specificity implies that a high proportion of patients without nerve root compromise have a positive SNRB result.

For peer review only

Table 1 Details of included studies

Author (Year) Country	N analysed/ N recruited	Description of included patients	Details of previous surgery	Needle level	Anaesthetic details	Guided method	Needle provocation	Number of control injections	Time to pain measurement
Within patient case-control studies									
Yeom[27] (2008) NR	47/83	Established single-level radiculopathy. Concordant imaging & clinical findings.	No history of lumbar surgeries.	L3, L4, L5, S1	1ml of 2% Lidocaine	Fluoroscopy	No	1 or 2	30 min
North[32] (1996) USA	33/33	Established sciatica with or without low back pain. History of nerve root compression or imaging findings of ongoing nerve root compression.	48% history of root compression corrected surgically.	L5, S1	3ml of 0.5% Bupivacaine	Fluoroscopy	Yes	3	Every 15 min for 3 hours
Prospective diagnostic cohort studies									
Schutz[31] (1973) Canada	15/23	Current sciatica symptoms.	Unclear if patients included in analysis had previous surgeries.	NR	1ml of Procaine (concentration NR)	Guided but method NR	Yes	1 or 2	Immediate
Retrospective diagnostic cohort studies									
Sasso[29] (2005) USA	83/83	Cervical or lumbar radiculopathy. Discordant imaging & clinical findings.	Unclear how many previous lumbar surgeries.	NR	0.5-0.7ml of 2% Lidocaine	Fluoroscopy	Yes	NR	Immediate
Dooley[32] (1988) Canada	62/73	Radicular pain & previous nerve root infiltration.	32 >=1 previous surgery, 3 had 4 surgeries.	L3, L4, L5, S1	1ml of 1% Mepivacaine or Lidocaine	Fluoroscopy	Yes	NR	Immediate
Williams[28] (2015) UK	96/100	Presumed radicular leg pain. Discordant clinical & imaging findings.	NR	L1, L3, L4, L5, S1	2 mL of 1% Lidocaine and 0.5 to 1 mL of Iopamidol	Fluoroscopy	Yes	NR	Immediate

Abbreviations: DRGB, dorsal root ganglion block; NR, not reported; MRI, Magnetic resonance imaging; SNRB, selective nerve root block.

For peer review only - http://bmjopen.bmj.com/site/about/guidelines.xhtml

Table 2 Diagnostic accuracy results

Author (year)	Threshold	Reference standard	TP	FN	Sensitivity % (95% CI)	TN	FP	Specificity % (95% CI)
Within-patient case-control studies								
Yeom[27] (2008)	70% pain relief – several other thresholds also evaluated.	Concordant symptoms and imaging evidence of compression (case injections) or no symptoms or imaging evidence of compression (control injections).	27	20	57 (43, 70)	50	8	86 (75, 93)
North[32] (1996)	50% reduction in baseline pain following block.	Concordant symptoms and imaging evidence of compression (case injections) or no symptoms or imaging evidence of compression (control injections).	30	3	91 (76, 97)	8	25	24 (12, 41)
Diagnostic cohort studies								
Schutz[31] (1973)	100% pain relief. Full trunk flexion and straight leg raising possible.	Intraoperative findings.	12	0	100 (76, 100)	1	2	33 (6, 79)
Sasso[29] (2005)	Visual Analog Scale score 0-1 & immediate relief of >95% pain	Outcome 12 months following surgery	71	3	96 (89, 99)	5	4	56 (27, 81)
Dooley[32] (1988)	Pain relief	Intraoperative surgical confirmation of root pathology	46	4	92 (81, 98)	2	1	67 (9, 99)
		Outcome following surgery (follow up range 24-36 months)	28	4	88 (71, 96)	2	19	10 (1, 30)
Williams[28] (2015)	Pain relief	Outcome 3 months following surgery (resolution of symptoms)	41	7	85 (72, 94)	2	10	17 (3, 48)

*Please note that the unit used in the within patient case-control studies is number of injections and some patients had two control injections at adjacent levels in addition to the affected nerve; in all other studies it is number of patients.
Abbreviations: TP, true positive; FN, false negative; TN, true negative; FP, false positive; CI, confidence interval.

For peer review only - http://bmjopen.bmj.com/site/about/guidelines.xhtml

Table 3 QUADAS-2 results

	RISK OF BIAS				APPLICABILITY CONCERNS		
**Author (year)**	**Patient selection**	**Index test**	**Reference standard**	**Flow and timing**	**Patient selection**	**Index test**	**Reference standard**
Within patient case control studies							
Yeom[27] (2008)	☹	☹	☹	☹	☹	☺	☹
North[31] (1996)	☹	☺	☹	☺	☹	☺	☹
Diagnostic cohort studies							
Sasso[29] (2005)	☺	☺	☹	☹	☺	☺	☺
Schutz[31] (1973)	?	☺	☹	☹	?	☺	☹
Dooley[32] (1988)	☺	☺	☹	☹	☺	☺	☺
Williams[28] (2015)	☺	?	☹	☹	☺	☺	☺

☺ **Low risk/ concern; ☹ High risk/ concern; ? Unclear risk/concern**

Adverse events review

Eight studies assessed complications and/or adverse events (Supplementary Table 2).[28 30 33-38] Two were diagnostic cohorts,[28 30] one was a randomized controlled trial[34] and five were case series.[33 35-38] Only one reported the complications of SNRBs in the lumbar spine as the primary outcome.[33] Five studies reported that there were no complications. Tajima et al. reported aggravated pain in the lower extremity for 1-2 days following selective radiculography and block in 4 (3.8%) patients.[38] The largest study reported that minor and transient complications were encountered in 98 of the 1777 total patient visits (during which 2217 injections were delivered to 1203 patients), giving an overall per patient visit complication rate of 5.5%.[33] Complications occurred in 134 of the 2217 total injections (6% complication rate per injection). There were no major or permanent complications resulting from SNRB in this large case series.

For peer review only - http://bmjopen.bmj.com/site/about/guidelines.xhtml

DISCUSSION

Despite the longstanding use of SNRB to help in the selection of patients who might benefit from surgery and in guiding the surgical approach, few studies have estimated its diagnostic accuracy. Our systematic review identified six studies, all at high risk of bias. Many were at risk of verification bias, because patients with positive SNRB were more likely to undergo surgery than those testing negative. There was substantial variation in estimates of sensitivity and specificity across studies. Based on the three cohort studies that used post-surgery outcomes as the reference standard, the summary sensitivity was 90.9% (83.1% - 95.3%) and summary specificity was 22.0% (7.4% - 49.9%). SNRB is a safe test with a low risk of clinically significant complications, but it remains unclear whether the additional diagnostic information it provides, improves patient outcomes or justifies the cost of the test.

Extensive literature searches were conducted in an attempt to locate all relevant studies. These included electronic searches in a wide variety of databases, scanning the references of included studies and previous systematic reviews. Diagnostic accuracy studies are difficult to identify from electronic databases as there are no specific indexing terms. Therefore, very sensitive searches were carried out to ensure that relevant studies were not missed. It is unlikely that any relevant published studies have been missed, although it is possible that some unpublished studies were not identified. The small number of primary diagnostic accuracy studies included in our review, all had methodological limitations. Due to the small number of studies, we were unable to explore the value of SNRB in potentially important patient subgroups, such as those with suspected multi-level radiculopathy.

Four previous systematic reviews of the diagnostic utility of SNRB in patients whose pain was of spinal origin have been reported.[15-18] The two earlier reviews had positive interpretations of the data and concluded that there was moderate evidence for SNRB in the "pre-operative evaluation of patients with negative or inconclusive imaging studies, but with clinical findings of nerve root irritation".[16 18] More recent reviews, however, concluded that there was limited evidence for the accuracy of SNRB as a diagnostic tool.[15 17] Our update review shows similar results. We found limited evidence which was of low methodological quality indicating that the diagnostic accuracy of SNRB is uncertain and that specificity in particular may be low. The differences in interpretation between our review and those conducted previously may be partly due to the smaller number of primary studies included in our review. We used rigorous eligibility criteria, which excluded studies with mixed cervical and

For peer review only - http://bmjopen.bmj.com/site/about/guidelines.xhtml

lumbar spine pathology and studies where there was insufficient data to construct estimates of sensitivity and specificity.

For centres that currently rely on SNRB for diagnostic information to help decide whether, or at which level, to perform lumbar decompressive surgery, it is vital that better evidence is generated. Moreover, according to Hospital Episodes Statistics (HES), which contains records of all admissions, appointments and attendances for patients at NHS hospitals in England, 58,399 injections of therapeutic substance around spinal nerve root took place from 01 April 2016 to 31 March 2017.[8] Due to the granularity of HES data, it is not possible to tell how many of these injections were diagnostic lumbar SNRB. Nevertheless, the number is substantial, and it is therefore apparent that the community of spinal surgeons has a responsibility to generate robust evidence for the use of diagnostic SNRBs. A methodologically ideal diagnostic accuracy study is unlikely to be clinically acceptable as it would require all patients, including those with negative SNRB findings, to undergo surgery. Furthermore, while diagnostic accuracy studies can explore whether SNRB accurately predicts surgical outcomes, they cannot answer the more fundamental question of whether SNRB improves surgical decisions and patient outcomes. Much better evidence would be provided by a trial randomising patients who are being considered for surgery but have discordant or equivocal clinical and imaging findings of nerve root compression to receive a diagnostic SNRB or to have management based on clinical and imaging findings alone. Given the lack of high quality evidence on the diagnostic accuracy of SNRB, we believe that such a trial would be ethically acceptable and would help patients, clinicians and health care payers decide whether SNRB can improve patient outcomes by targeting surgery at those most likely to benefit.

Finally, it should be mentioned that this systematic review did not consider the use of SNRBs as a therapeutic option for patients with radicular pain due to a prolapsed lumbar intervertebral disc. The most recent NICE guidance concluded that the evidence for both image guided and non-image guided injections for patients with acute and severe sciatica was mostly low or moderate.[8] However, the guidance recommends that an injection of local anaesthetic and steroid should be considered in acute, severe sciatica where patients would otherwise be offered surgery. The NERVES randomised trial, which enrolled patients in 12 NHS hospitals, aimed to compare surgical microdiscectomy versus SNRB in patients with sciatica of at least 6 weeks' duration secondary to a prolapsed intervertebral disc. The results of this trial, which is currently in follow-up, will elucidate the role of

For peer review only - http://bmjopen.bmj.com/site/about/guidelines.xhtml

SNRB as a therapeutic but not diagnostic option. Hence, it is important that consideration is given to a trial of diagnostic SNRB as outlined above.

CONCLUSIONS

There is no high-quality evidence on the diagnostic accuracy of SNRB in patients with radiculopathy and discordant or equivocal imaging findings. The evidence that is available suggests that the specificity of SNRB is low. As there is no adequate reference standard for determining the diagnostic accuracy of SNRB, future research should focus on randomised controlled trials to evaluate whether SNRB improves the process of care or patient outcomes.

For peer review only

For peer review only - http://bmjopen.bmj.com/site/about/guidelines.xhtml

Funding

PW and ME are funded by a National Institute for Health Research Collaboration for Leadership in Applied Health Research and Care West (NIHR CLAHRC West). AS is funded by an NIHR Systematic Review Fellowship (RM-SR-2017-08-012). AK is supported by a Clinical Lectureship, School of Clinical Medicine, University of Cambridge. RB, RL, PW and WH were funded by a National Institute for Health Research Health Technology Assessment programme grant (project number 09/111/01).

Competing interests

None declared

Author contributions

RB conducted the reviews of diagnostic accuracy and adverse events, conducted analyses and completed the first draft of the manuscript. ME updated the review, including abstract and full text screening, data extraction, risk of bias assessment, analysis and updated the draft of the manuscript. AS updated the review, including abstract and full text screening and checking of data extraction and risk of bias assessment. RL contributed to the conception and design of the study, provided clinical expertise in neurosurgery, helped with acquisition of data for the service evaluation of SNRB and critically revised the manuscript. AK provided clinical expertise in neurosurgery and critically revised the manuscript. PW contributed to the conception and design of the study, supervised conduct of reviews of diagnostic accuracy and adverse events, and critically revised the manuscript. WH was principal investigator on the project, contributed to the conception and design of the study, supervised conduct of reviews of diagnostic accuracy and adverse events and critically revised the manuscript. All authors approved the manuscript for publication.

Acknowledgements

The authors wish to thank Margaret Burke for her advice in developing and implementing the search Strategy, Catherine Jameson for implementing the original searches and reviews and Alison Richards for conducting the update searches. This research was supported by the National Institute for Health Research (NIHR) Collaboration for Leadership in Applied Health Research and Care West (NIHR CLAHRC West) and the

For peer review only

NIHR Health Technology Assessment programme. The views expressed in this article are those of the author(s) and not necessarily those of the NHS, the NIHR, or the Department of Health and Social Care.

For peer review only

For peer review only - http://bmjopen.bmj.com/site/about/guidelines.xhtml

REFERENCES

1. Maniadakis N, Gray A. The economic burden of back pain in the UK. *Pain* 2000;84(1):95-103. doi: S0304-3959(99)00187-6 [pii] [published Online First: 1999/12/22]
2. Jacobs WC, van Tulder M, Arts M, et al. Surgery versus conservative management of sciatica due to a lumbar herniated disc: a systematic review. *Eur Spine J* 2011;20(4):513-22. doi: 10.1007/s00586-010-1603-7 [published Online First: 2010/10/16]
3. Weinstein JN, Tosteson TD, Lurie JD, et al. Surgical versus nonsurgical therapy for lumbar spinal stenosis. *N Engl J Med* 2008;358(8):794-810. doi: 10.1056/NEJMoa0707136 [published Online First: 2008/02/22]
4. Weinstein JN, Tosteson TD, Lurie JD, et al. Surgical vs nonoperative treatment for lumbar disk herniation: the Spine Patient Outcomes Research Trial (SPORT): a randomized trial. *JAMA* 2006;296(20):2441-50. doi: 10.1001/jama.296.20.2441 [published Online First: 2006/11/23]
5. Peul WC, van Houwelingen HC, van den Hout WB, et al. Surgery versus prolonged conservative treatment for sciatica. *N Engl J Med* 2007;356(22):2245-56. doi: 10.1056/NEJMoa064039 [published Online First: 2007/06/01]
6. Peul WC, van den Hout WB, Brand R, et al. Prolonged conservative care versus early surgery in patients with sciatica caused by lumbar disc herniation: two year results of a randomised controlled trial. *British Medical Journal* 2011;336(7657)
7. Atlas SJ, Keller RB, Chang Y, et al. Surgical and nonsurgical management of sciatica secondary to a lumbar disc herniation: five-year outcomes from the Maine Lumbar Spine Study. *Spine (Phila Pa 1976)* 2001;26(10):1179-87. [published Online First: 2001/06/20]
8. The National Institute for Health and Care Excellence (NICE). Low back pain and sciatica in over 16s: assessment and management (NG59). 2016
9. Parker SL, Mendenhall SK, Godil SS, et al. Incidence of Low Back Pain After Lumbar Discectomy for Herniated Disc and Its Effect on Patient-reported Outcomes. *Clin Orthop Relat Res* 2015;473(6):1988-99. doi: 10.1007/s11999-015-4193-1 [published Online First: 2015/02/20]
10. Baber Z, Erdek MA. Failed back surgery syndrome: current perspectives. *J Pain Res* 2016;9:979-87. doi: 10.2147/JPR.S92776 [published Online First: 2016/11/18]
11. Deyo RA, Diehl AK. Lumbar Spine Films in Primary Care - Current Use and Effects of Selective Ordering Criteria. *Journal of General Internal Medicine* 1986;1(1):20-25. doi: 10.1007/Bf02596320
12. Vroomen PC, de Krom MC, Wilmink JT, et al. Diagnostic value of history and physical examination in patients suspected of lumbosacral nerve root compression. *J Neurol Neurosurg Psychiatry* 2002;72(5):630-4. [published Online First: 2002/04/24]
13. Rubinstein SM, van Tulder M. A best-evidence review of diagnostic procedures for neck and low-back pain. *Best Pract Res Clin Rheumatol* 2008;22(3):471-82. doi: 10.1016/j.berh.2007.12.003 [published Online First: 2008/06/04]
14. Boswell MV, Trescot AM, Datta S, et al. Interventional techniques: evidence-based practice guidelines in the management of chronic spinal pain. *Pain Physician* 2007;10(1):7-111. [published Online First: 2007/01/27]
15. Beynon R, Hawkins J, Laing R, et al. The diagnostic utility and cost-effectiveness of selective nerve root blocks in patients considered for lumbar decompression surgery: a systematic review and economic model. *Health Technol Assess* 2013;17(19):1-88, v-vi. doi: 10.3310/hta17190 [published Online First: 2013/05/16]
16. Datta S, Everett CR, Trescot AM, et al. An updated systematic review of the diagnostic utility of selective nerve root blocks. *Pain Physician* 2007;10(1):113-28. [published Online First: 2007/01/27]
17. Datta S, Manchikanti L, Falco FJ, et al. Diagnostic utility of selective nerve root blocks in the diagnosis of lumbosacral radicular pain: systematic review and update of current evidence. *Pain Physician* 2013;16(2 Suppl):SE97-124. [published Online First: 2013/05/03]
18. Everett CR, Shah RV, Sehgal N, et al. A systematic review of diagnostic utility of selective nerve root blocks. *Pain Physician* 2005;8(2):225-33. [published Online First: 2006/07/20]
19. Leeflang MMG, Scholten RJPM, Rutjes AWS, et al. Use of methodological search filters to identify diagnostic accuracy studies can lead to the omission of relevant studies. *J Clin Epidemiol* 2006;59(3):234-40. doi: 10.1016/j.jclinepi.2005.07.014

For peer review only - http://bmjopen.bmj.com/site/about/guidelines.xhtml

20. Whiting P, Westwood M, Beynon R, et al. Inclusion of methodological filters in searches for diagnostic test accuracy studies misses relevant studies. *J Clin Epidemiol* 2011;64(6):602-7. doi: 10.1016/j.jclinepi.2010.07.006 [published Online First: 2010/11/16]
21. Beynon R, Leeflang MM, McDonald S, et al. Search strategies to identify diagnostic accuracy studies in MEDLINE and EMBASE. *Cochrane Database Syst Rev* 2013(9):MR000022. doi: 10.1002/14651858.MR000022.pub3 [published Online First: 2013/09/12]
22. Whiting PF, Rutjes AW, Westwood ME, et al. QUADAS-2: a revised tool for the quality assessment of diagnostic accuracy studies. *Ann Intern Med* 2011;155(8):529-36. doi: 10.7326/0003-4819-155-8-201110180-00009 [published Online First: 2011/10/19]
23. StataCorp. Stata Statistical Software: Release 15. *College Station, TX: StataCorp LLC* 2017
24. DerSimonian R, Laird N. Meta-analysis in clinical trials. *Control Clin Trials* 1986;7(3):177-88. [published Online First: 1986/09/01]
25. Harbord RM, Whiting P. metandi: Meta-analysis of diagnostic accuracy using hierarchical logistic regression. *Stata Journal* 2009;9(2):211-29.
26. McInnes MDF, Moher D, Thombs BD, et al. Preferred Reporting Items for a Systematic Review and Meta-analysis of Diagnostic Test Accuracy Studies: The PRISMA-DTA Statement. *JAMA* 2018;319(4):388-96. doi: 10.1001/jama.2017.19163 [published Online First: 2018/01/25]
27. Yeom JS, Lee JW, Park KW, et al. Value of diagnostic lumbar selective nerve root block: a prospective controlled study. *AJNR Am J Neuroradiol* 2008;29(5):1017-23. doi: 10.3174/ajnr.A0955 [published Online First: 2008/02/15]
28. Schutz H, Lougheed WM, Wortzman G, et al. Intervertebral nerve-root in the investigation of chronic lumbar disc disease. *Can J Surg* 1973;16(3):217-21. [published Online First: 1973/05/01]
29. Dooley JF, McBroom RJ, Taguchi T, et al. Nerve root infiltration in the diagnosis of radicular pain. *Spine (Phila Pa 1976)* 1988;13(1):79-83. [published Online First: 1988/01/01]
30. Williams AP, Germon T. The value of lumbar dorsal root ganglion blocks in predicting the response to decompressive surgery in patients with diagnostic doubt. *Spine J* 2015;15(3 Suppl):S44-9. doi: 10.1016/j.spinee.2015.01.004 [published Online First: 2015/01/13]
31. Sasso RC, Maradaeg K, Nordmann D, et al. Selective nerve root injections can predict surgical outcome for lumbar and cervical radiculopathy - Comparison to magnetic resonance imaging. *Journal of Spinal Disorders & Techniques* 2005;18(6):471-78. doi: 10.1097/01.bsd.0000146761.36658.45
32. North RB, Kidd DH, Zahurak M, et al. Specificity of diagnostic nerve blocks: A prospective, randomized study of sciatica due to lumbosacral spine disease. *Pain* 1996;65(1):77-85. doi: 10.1016/0304-3959(95)00170-0
33. Stalcup ST, Crall TS, Gilula L, et al. Influence of needle-tip position on the incidence of immediate complications in 2,217 selective lumbar nerve root blocks. *Spine J* 2006;6(2):170-6. doi: 10.1016/j.spinee.2005.08.009 [published Online First: 2006/03/07]
34. Ghahreman A, Ferch R, Bogduk N. The efficacy of transforaminal injection of steroids for the treatment of lumbar radicular pain. *Pain Med* 2010;11(8):1149-68. doi: 10.1111/j.1526-4637.2010.00908.x [published Online First: 2010/08/14]
35. Jonsson B, Stromqvist B, Annertz M, et al. Diagnostic lumbar nerve root block. *J Spinal Disord* 1988;1(3):232-5. [published Online First: 1988/01/01]
36. Ng LC, Sell P. Outcomes of a prospective cohort study on peri-radicular infiltration for radicular pain in patients with lumbar disc herniation and spinal stenosis. *Eur Spine J* 2004;13(4):325-9. doi: 10.1007/s00586-003-0649-1 [published Online First: 2004/01/10]
37. Quinn SF, Murtagh FR, Chatfield R, et al. CT-guided nerve root block and ablation. *AJR Am J Roentgenol* 1988;151(6):1213-6. doi: 10.2214/ajr.151.6.1213 [published Online First: 1988/12/01]
38. Tajima T, Furukawa K, Kuramochi E. Selective lumbosacral radiculography and block. *Spine (Phila Pa 1976)* 1980;5(1):68-77. [published Online First: 1980/01/01]

For peer review only - http://bmjopen.bmj.com/site/about/guidelines.xhtml

Figure legends

Figure 1 Flowchart of diagnostic study selection process

Figure 2 ROC plot displaying diagnostic accuracy results of included studies. Abbreviations: PS, Post-surgical reference standard; IO, Intraoperative reference standard; CI, Control injection reference standard.

For peer review only

For peer review only - http://bmjopen.bmj.com/site/about/guidelines.xhtml

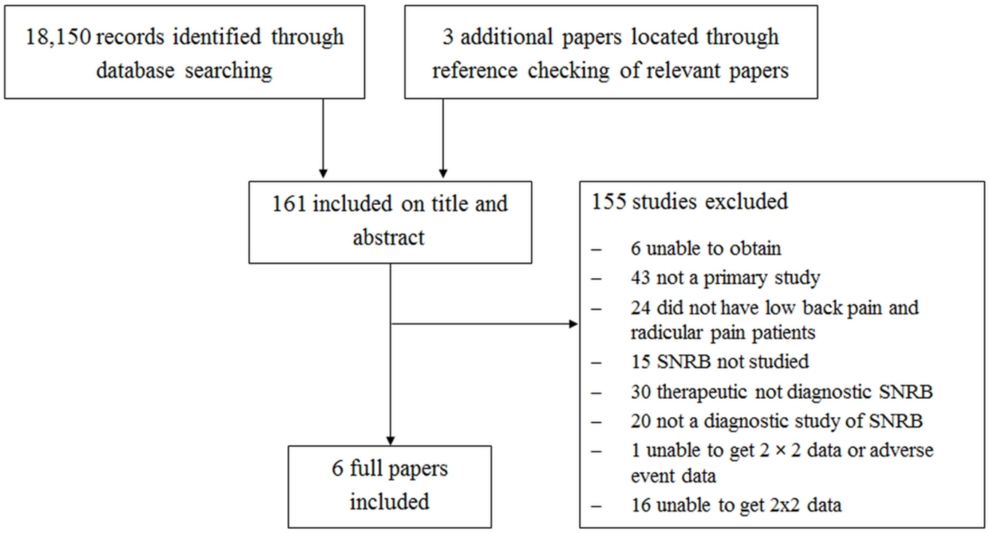

Flowchart of diagnostic study selection process

165x90mm (300 x 300 DPI)

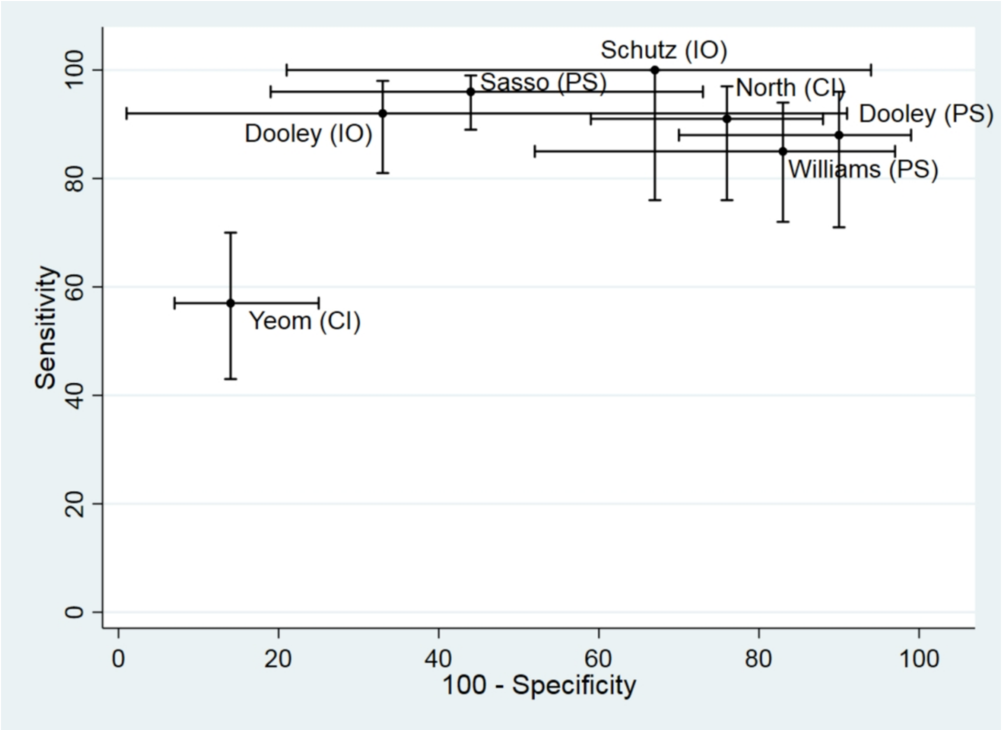

ROC plot displaying diagnostic accuracy results of included studies. Abbreviations: PS, Post-surgical reference standard; IO, Intraoperative reference standard; CI, Control injection reference standard.

123x90mm (300 x 300 DPI)

Supplementary Table 1 Details of included studies

Author (Year) Country	N analysed/N recruited	Inclusion criteria	Description of included patients	Details of previous surgery	Needle level	Anaesthetic details	Guided method	Needle provocation	Number of control injections	Time to pain measurement
Within patient case-control studies										
Yeom[26] (2008) NR	47/83	Patients due to undergo a lumbar spine operation with single-level, unilateral lumbosacral radiculopathy confirmed by clinical, radiographic & MRI findings.	Patients with established pure radiculopathy from a single level. Affected roots were L4 in 3, L5 in 31, S1 in 13. Concordant imaging & clinical findings.	No history of lumbar surgeries	L3, L4, L5, S1	1ml of 2% Lidocaine	Fluoroscopy	No	1 or 2	30 minutes
North[29] (1996) USA	33/33	Patients with sciatica with or without low back pain, attributed to spinal pathology.	Established sciatica patients with or without low back pain. All had L5 or S1 radiculopathy. 52% had diagnostic imaging findings of ongoing nerve root compression. The remaining 48% had a well-documented history of root compression which had been corrected surgically.	48% history of root compression corrected surgically.	L5, S1	3ml of 0.5% Bupivacaine	Fluoroscopy	Yes	3	Every 15 minutes for 3 hours
Prospective diagnostic cohort studies										
Schutz[31] (1973) Canada	15/23	Patients with current sciatica.	Patients with sciatica. Investigation undertaken only at a time when sciatica symptoms actually present.	1 patient had previous surgery, unsuccessful SNRB & excluded from analysis. Unclear if patients	NR	1ml of Procaine (concentrati	Guided but method not reported	Yes	1 or 2	Immediate

For peer review only - http://bmjopen.bmj.com/site/about/guidelines.xhtml

Author (Year) Country	N analysed/N recruited	Inclusion criteria	Description of included patients	Details of previous surgery	Needle level	Anaesthetic details	Guided method	Needle provocation	Number of control injections	Time to pain measurement
				included in analysis had previous surgeries.		on not reported)				
Retrospective diagnostic cohort studies										
Sasso[29] (2005) USA	83/83	Patients who underwent SNRB, MRI & nerve root decompression surgery and had a follow-up evaluation >12 months post surgery	Patients with cervical or lumbar radiculopathy. Discordant imaging and clinical findings	Unclear how many previous lumbar surgeries. 20 patients with cervical or lumbar symptoms had previous surgery	NR	0.5-0.7ml of 2% Lidocaine	Fluoroscopy	Yes	NR	Immediate
Dooley[32] (1988) Canada	62/73	Patients who underwent nerve root infiltration	Patients with radicular pain who underwent nerve root infiltration	32 >=1 previous surgery, 3 had 4 surgeries.	L3, L4, L5, S1	1ml of 1% Mepivacaine or Lidocaine	Fluoroscopy	Yes	NR	Immediate
Williams[28] (2015) UK	96/100	Patients who underwent diagnostic lumbar DRGB (identified retrospectively)	Patients with presumed radicular leg pain with significant diagnostic uncertainty from the patient's presenting history, examination and imaging as to whether lumbosacral nerve root compression was indeed responsible.	NR	L1, L3, L4, L5, S1	2 mL of 1% Lidocaine and 0.5 to 1 mL of Iopamidol	Fluoroscopy	Yes	NR	Immediate

Abbreviations: DRGB, dorsal root ganglion block; NR, not reported; MRI, Magnetic resonance imaging; SNRB, selective nerve root block.

For peer review only

Supplementary Table 2 Patient characteristics of studies reporting on adverse events following SNRB

Author (year) Country	N analysed/ N recruited	Inclusion criteria	Needle tip position	Needle levels	Anaesthetic details	Guided method	Needle provocation used?	Adverse events
Case-series								
Stalcup[32] (2006) USA	1203/ 1203	All adult patients who underwent a SNRB in the lumbar spine in a radiology department.	Adjacent or into the intervertebral foramen.	NR	1-2ml of 0.25% Bupivacaine	Fluoroscopy	NR	Numbers given in injections: Leg weakness n=77; Pain increased n=51; Other n=6; Total n=134
Ng[35] (2004) UK	117/ 125	Consecutive patients with clinical evidence of unilateral radicular pain that lasted despite at least 6 weeks of conservative management, MRI confirmation of nerve root compression secondary to lumbar disc herniation or peripheral degenerative spinal stenosis.	Superiorly to pedicle, medially to nerve and laterally to vertebral body.	NR	2ml of 0.25% Bupivacaine	Assumed Fluoroscopy	NR	No adverse events
Jonsson[34] (1988) Sweden	78/ 78	Patients with unilateral sciatic pain, considered for surgery. Sciatic pain but normal findings on myelography, CT and/or MRI.	Just lateral to the opening of the intervertebral foramen.	L4, L5, S1	3-6ml of Carbocaine (% NR)	Fluoroscopy	NR	No adverse events.
Quinn[36] (1988) USA	33/ 33	Patients with a herniated disc or foraminal stenosis (n=2) as identified by CT or MRI.	An attempt was made to pierce the nerve or to have the needle tip within 1-2mm of the nerve.	NR	2.5-5ml of 1% Lidocaine or 0.5% Bupivacaine	CT	Yes	No adverse events.
Tajima[37] (1980) Japan	106/ 106	Patients with radicular symptoms undergoing lumbosacral radiculography and block who had lumbosacral diseases.	Approx 4cm lateral to upper margin of lumbar spinous process corresponding to nerve root to be radiographed.	L4, L5, S1	3ml of 1% Lidocaine	x-ray	Yes	Pain in the lower extremity was aggravated for 1-2 days

For peer review only - http://bmjopen.bmj.com/site/about/guidelines.xhtml

								following selective radiculography and block in 4 patients. There was no other complication.
Diagnostic cohort study								
*Schutz[31] (1973) Canada	15/ 23	Patients with current sciatica.	Superior level of intervertebral foramen. Introduced about 2" from the midline.	NR	1ml Procaine	Guided but method not reported	Yes	No adverse events.
*Williams[28] (2015) UK	96/100	Patients with presumed radicular leg pain who underwent diagnostic lumbar DRGB (identified retrospectively).	Inserted from a paraspinal entry point and advanced to the superoanterior margin of the intervertebral foramen of the targeted level.	L1,L3, L4,L5 & S1	2 mL of 1% Lidocaine and 0.5 to 1 mL of Iopamidol	Fluoroscopy	Yes	No adverse events.
Randomized controlled trial								
Ghahreman[33] (2010) Australia	27/ 150	Adult patients with lower limb radiculopathy; limitation of straight-leg raise to <30º; disc herniation on CT or MRI. Considered for surgery. Only data for single arm of trial in which patients received anaesthetic was included in the current review.	Placed in the intervertebral foramen of the target level.	L2,L3, L4,L5 & S1	2ml of 0.5% Bupivacaine	Assumed Fluoroscopy	NR	No complications occurred that could be attributed to the treatment.

*Included in diagnostic accuracy systematic review

Abbreviations: CT, computerised tomography; DRGB, dorsal root ganglion block; MRI, magnetic resonance imaging; NR, not reported; SNRB, selective nerve root block.

For peer review only

PRISMA-DTA Checklist

Section/topic	#	PRISMA-DTA Checklist Item	Reported on page #
**TITLE / ABSTRACT**			
Title	1	Identify the report as a systematic review (+/- meta-analysis) of diagnostic test accuracy (DTA) studies.	P1
Abstract	2	Abstract: See PRISMA-DTA for abstracts.	P2
**INTRODUCTION**			
Rationale	3	Describe the rationale for the review in the context of what is already known.	P4
Clinical role of index test	D1	State the scientific and clinical background, including the intended use and clinical role of the index test, and if applicable, the rationale for minimally acceptable test accuracy (or minimum difference in accuracy for comparative design).	P4
Objectives	4	Provide an explicit statement of question(s) being addressed in terms of participants, index test(s), and target condition(s).	P5
**METHODS**			
Protocol and registration	5	Indicate if a review protocol exists, if and where it can be accessed (e.g., Web address), and, if available, provide registration information including registration number.	NA
Eligibility criteria	6	Specify study characteristics (participants, setting, index test(s), reference standard(s), target condition(s), and study design) and report characteristics (e.g., years considered, language, publication status) used as criteria for eligibility, giving rationale.	P6
Information sources	7	Describe all information sources (e.g., databases with dates of coverage, contact with study authors to identify additional studies) in the search and date last searched.	P6
Search	8	Present full search strategies for all electronic databases and other sources searched, including any limits used, such that they could be repeated.	P6
Study selection	9	State the process for selecting studies (i.e., screening, eligibility, included in systematic review, and, if applicable, included in the meta-analysis).	P6
Data collection process	10	Describe method of data extraction from reports (e.g., piloted forms, independently, in duplicate) and any processes for obtaining and confirming data from investigators.	P6
Definitions for data extraction	11	Provide definitions used in data extraction and classifications of target condition(s), index test(s), reference standard(s) and other characteristics (e.g. study design, clinical setting).	P7
Risk of bias and applicability	12	Describe methods used for assessing risk of bias in individual studies and concerns regarding the applicability to the review question.	P7
Diagnostic accuracy measures	13	State the principal diagnostic accuracy measure(s) reported (e.g. sensitivity, specificity) and state the unit of assessment (e.g. per-patient, per-lesion).	P7
Synthesis of results	14	Describe methods of handling data, combining results of studies and describing variability between studies. This could include, but is not limited to: a) handling of multiple definitions of target condition. b) handling of multiple thresholds of test positivity, c) handling multiple index test readers, d) handling of indeterminate test results, e) grouping and comparing tests, f) handling of different reference standards	P7

For peer review only - http://bmjopen.bmj.com/site/about/guidelines.xhtml

PRISMA-DTA Checklist

Section/topic	#	PRISMA-DTA Checklist Item	Reported on page #
Meta-analysis	D2	Report the statistical methods used for meta-analyses, if performed.	P7
Additional analyses	16	Describe methods of additional analyses (e.g., sensitivity or subgroup analyses, meta-regression), if done, indicating which were pre-specified.	P7
**RESULTS**			
Study selection	17	Provide numbers of studies screened, assessed for eligibility, included in the review (and included in meta-analysis, if applicable) with reasons for exclusions at each stage, ideally with a flow diagram.	P8
Study characteristics	18	For each included study provide citations and present key characteristics including: a) participant characteristics (presentation, prior testing), b) clinical setting, c) study design, d) target condition definition, e) index test, f) reference standard, g) sample size, h) funding sources	P8,9,10
Risk of bias and applicability	19	Present evaluation of risk of bias and concerns regarding applicability for each study.	P8,9,12
Results of individual studies	20	For each analysis in each study (e.g. unique combination of index test, reference standard, and positivity threshold) report 2x2 data (TP, FP, FN, TN) with estimates of diagnostic accuracy and confidence intervals, ideally with a forest or receiver operator characteristic (ROC) plot.	P11
Synthesis of results	21	Describe test accuracy, including variability; if meta-analysis was done, include results and confidence intervals.	P9
Additional analysis	23	Give results of additional analyses, if done (e.g., sensitivity or subgroup analyses, meta-regression; analysis of index test: failure rates, proportion of inconclusive results, adverse events).	P9,12
**DISCUSSION**			
Summary of evidence	24	Summarize the main findings including the strength of evidence.	P13
Limitations	25	Discuss limitations from included studies (e.g. risk of bias and concerns regarding applicability) and from the review process (e.g. incomplete retrieval of identified research).	P13,14
Conclusions	26	Provide a general interpretation of the results in the context of other evidence. Discuss implications for future research and clinical practice (e.g. the intended use and clinical role of the index test).	P14,15
**FUNDING**			
Funding	27	For the systematic review, describe the sources of funding and other support and the role of the funders.	P16

Adapted From: McInnes MDF, Moher D, Thombs BD, McGrath TA, Bossuyt PM, The PRISMA-DTA Group (2018). Preferred Reporting Items for a Systematic Review and Meta-analysis of Diagnostic Test Accuracy Studies: The PRISMA-DTA Statement. JAMA. 2018 Jan 23;319(4):388-396. doi: 10.1001/jama.2017.19163.

For more information, visit: **www.prisma-statement.org**.

For peer review only - http://bmjopen.bmj.com/site/about/guidelines.xhtml

BMJ Open

The utility of diagnostic selective nerve root blocks in the management of patients with lumbar radiculopathy: a systematic review

Journal:	*BMJ Open*
Manuscript ID	bmjopen-2018-025790.R1
Article Type:	Research
Date Submitted by the Author:	07-Jan-2019
Complete List of Authors:	Beynon, Rebecca; University of Bristol, Population Health Sciences, Bristol Medical School<br>Elwenspoek, Martha; University Hospitals Bristol NHS Foundation Trust, The National Institute for Health Research Collaboration for Leadership in Applied Health Research and Care West (NIHR CLAHRC West); University of Bristol, Population Health Sciences, Bristol Medical School<br>Sheppard, Athena; University of Bristol, Population Health Sciences, Bristol Medical School; University Hospitals Bristol NHS Foundation Trust, The National Institute for Health Research Collaboration for Leadership in Applied Health Research and Care West (NIHR CLAHRC West)<br>Higgins, John; Addenbrooke's Hospital, Radiology<br>Kolias, Angelos; Addenbrooke's Hospital & University of Cambridge, Division of Neurosurgery, Department of Clinical Neurosciences<br>Laing, Rodney; Addenbrooke's Hospital & University of Cambridge, Division of Neurosurgery, Department of Clinical Neurosciences<br>Whiting, Penny; University of Bristol, Population Health Sciences, Bristol Medical School; University Hospitals Bristol NHS Foundation Trust, The National Institute for Health Research Collaboration for Leadership in Applied Health Research and Care West (NIHR CLAHRC West)<br>Hollingworth, William; University of Bristol, Population Health Sciences, Bristol Medical School
**Primary Subject Heading**:	Evidence based practice
Secondary Subject Heading:	Surgery, Diagnostics, Rheumatology
Keywords:	diagnostic accuracy, selective nerve root blocks, lumbar radiculopathy, low back pain, lumbar decompression surgery

SCHOLARONE™
Manuscripts

The utility of diagnostic selective nerve root blocks in the management of patients with lumbar radiculopathy: a systematic review

Rebecca Beynon[2], Martha MC Elwenspoek[1,2], Athena Sheppard[1,2], Nicholas J Higgins[3], Angelos G Kolias[4], Rodney J Laing[4], Penny Whiting[1,2], William Hollingworth[2]

Affiliations:

1. The National Institute for Health Research Collaboration for Leadership in Applied Health Research and Care West (NIHR CLAHRC West), University Hospitals Bristol NHS Foundation Trust, Bristol, UK
2. Population Health Sciences, Bristol Medical School, University of Bristol, Bristol, UK
3. Department of Radiology, Addenbrooke's Hospital, Cambridge, UK
4. Division of Neurosurgery, Department of Clinical Neurosciences, Addenbrooke's Hospital & University of Cambridge, Cambridge, UK

Corresponding author:

Martha M C Elwenspoek

9th Floor, Whitefriars, Lewins Mead,

Bristol, BS1 2NT

Email: martha.elwenspoek@bristol.ac.uk

Tel: +44/0 117 3427275

Word count: 3181

For peer review only

For peer review only - http://bmjopen.bmj.com/site/about/guidelines.xhtml

ABSTRACT

Objective: Lumbar radiculopathy often manifests as pain in the lower back radiating into one leg (sciatica). Unsuccessful back surgery is associated with significant healthcare costs and risks to patients. This review aims to examine the diagnostic accuracy of selective nerve root blocks (SNRB) to identify patients most likely to benefit from lumbar decompression surgery.

Design: Systematic review of diagnostic test accuracy studies.

Eligibility criteria: Primary research articles using a patient population with low back pain and symptoms in the leg, SNRB administered under radiological guidance as index test, and any reported reference standard for the diagnosis of lumbar radiculopathy.

Information sources: MEDLINE (Ovid), MEDLINE In-Process & Other Non-Indexed Citations, EMBASE, Science Citation Index, Biosis, LILACS, Dissertation abstracts and NTIS from inception to 2018.

Methods: Risk of bias and applicability was assessed using the QUADAS-2 tool. We performed random effects logistic regression to meta-analyse studies grouped by reference standard.

Results: 6 studies (341 patients) were included in this review. All studies were judged at high risk of bias. There was substantial heterogeneity across studies in sensitivity (range 57-100%) and specificity (10-86%) estimates. Four studies were diagnostic cohort studies that used either intra-operative findings during surgery (pooled sensitivity: 93.5% [95% CI 84.0-97.6]; specificity: 50.0% [16.8- 83.2]) or 'outcome following surgery' as the reference standard (pooled sensitivity: 90.9% [83.1- 95.3]; specificity 22.0% [7.4- 49.9]). Two studies had a within-patient case-control study design, but results were not pooled because different types of control injections were used.

Conclusions: We found limited evidence which was of low methodological quality indicating that the diagnostic accuracy of SNRB is uncertain and that specificity in particular may be low. SNRB is a safe test with a low risk of clinically significant complications, but it remains unclear whether the additional diagnostic information it provides justifies the cost of the test.

Keywords: diagnostic accuracy of selective nerve root blocks (SNRB), lumbar radiculopathy, low back pain, lumbar decompression surgery.

For peer review only - http://bmjopen.bmj.com/site/about/guidelines.xhtml

ARTICLE SUMMARY

Strengths and limitations of this study

- Comprehensive synthesis of the current evidence on diagnostic accuracy of SNRB in lumbar radiculopathy.
- Extensive literature searches were conducted using several databases without restrictions on publication date, language, or study type, in an attempt to locate all relevant studies.
- We used rigorous eligibility criteria, which excluded studies with mixed cervical and lumbar spine pathology and studies where there was insufficient data to construct estimates of sensitivity and specificity.
- Only a small number of primary diagnostic accuracy studies could be included in our review and all had methodological limitations.
- Due to the small number of studies, we were unable to explore the value of SNRB in potentially important patient subgroups, such as those with suspected multi-level radiculopathy.

For peer review only - http://bmjopen.bmj.com/site/about/guidelines.xhtml

INTRODUCTION

In Western Europe, low back pain is the leading cause of disability and represents a high economic burden,[1] in particular due to production losses and cost of informal care.[1] In a subgroup of patients, low back pain is accompanied by pain radiating to a lower extremity in a radicular distribution (sciatic pain). Leg pain is one of the symptoms of lumbar radiculopathy (LR) but other symptoms, such as numbness, tingling, weakness, can also develop." LR can be the result of compressive or inflammatory disorders of the spinal nerve roots or a combination of these. Randomized trial evidence on the effectiveness of lumbar decompressive surgery in patients with radiculopathy and intervertebral disc herniation suggests that early surgery leads to faster pain relief, but longer-term effectiveness is less clear.[2-7] Current UK guidelines recommend spinal decompression surgery for patients with radicular pain when non-surgical treatments have not improved symptoms and radiological findings are consistent with physical examination.[8] However, surgery does not always resolve radicular pain and 5-36% of patients suffer from recurrent back and leg pain within 2 years post-surgery.[9] The main cause of unsuccessful back surgery is inaccurate diagnosis.[10] Improved diagnosis could help identify patients most likely to benefit from surgery and minimise the cost and risks associated with unsuccessful back surgery.

A timely and accurate diagnosis of the cause of low back pain and radicular pain is important, since it is occasionally an early symptom of serious systemic disease,[11] and an inaccurate diagnosis can lead to a cascade of costly, invasive and ineffective therapy. In most patients the diagnosis of radiculopathy, caused by nerve root compression, is made by correlation of symptoms, clinical signs, and imaging findings. However, neither clinical findings nor radiological imaging have perfect diagnostic accuracy.[12] When clinical and imaging findings are equivocal or discordant, uncertainty remains about the source of the symptoms and whether nerve root decompression will relieve symptoms. Additional diagnostic tests could help clinicians and patients to choose between surgical and conservative care or guide surgery in patients with suspected multi-level radiculopathy.

Diagnostic selective nerve root blocks (SNRB) inject local anaesthetic or other substances around spinal nerves under imaging guidance. Both provocative responses (replicating symptoms during needle placement) and analgesic responses (significant reduction of symptoms) to SNRB may be diagnostically useful in confirming or ruling out a given nerve root as the source of clinical symptoms. Some clinical guidelines and consensus

For peer review only - http://bmjopen.bmj.com/site/about/guidelines.xhtml

statements have endorsed the use of SNRB to identify the source of pain in patients with multilevel pathology and in the pre-operative evaluation of patients with a negative or inconclusive imaging study.[13 14] Over the last decade, several systematic reviews have investigated SNRB as diagnostic tool, covering the literature up to 2012.[15-18] However, evidence was scarce and of low quality and the diagnostic accuracy and reliability of SNRB remained unclear. We updated our previous systematic review to determine the diagnostic performance of SNRB in addition to clinical and imaging findings for identifying patients with lumbar radiculopathy who are good candidates for lumbar decompression surgery.[15] A secondary aim was to summarise evidence on the incidence of procedure related complications.

For peer review only

For peer review only - http://bmjopen.bmj.com/site/about/guidelines.xhtml

MATERIALS AND METHODS

Literature search

We updated the search from our previous review, searching all databases to March 2018. Our previous search aimed to identify published and unpublished studies by searching MEDLINE (Ovid), MEDLINE In-Process & Other Non-Indexed Citations, EMBASE, Science Citation Index, Biosis and LILACS (Latin American and Caribbean literature database), Dissertation abstracts and NTIS (National Technical Information Service) from inception to March 2018. Our search strategy combined terms for SNRB with terms for sciatica or radiculopathy (see Supplementary Search Strategy).[15] We did not use a methodological search filter to identify diagnostic accuracy studies as such filters result in the omission of relevant studies.[19-21] No language restrictions were applied. Attempts were made to identify further studies by examining the reference lists of all included articles.

Study selection

Studies were eligible for the diagnostic accuracy review if they included patients with low back pain and leg pain who underwent SNRB under imaging guidance. The studies needed to report sufficient data to construct a table detailing diagnostic accuracy (i.e. numbers of true negative, true positive, false positive, and false negative results) of the index test (SNRB) compared to any reported 'reference standard'. When we were unable to extract sufficient details from otherwise eligible studies we contacted study authors.

In diagnostic accuracy studies, the reference standard is typically a definitive test used to determine the true diagnosis, but no such definitive test exists for radicular pain due to nerve root compression. Therefore, most diagnostic studies used either intra-operative findings or post-surgical follow up as the reference standard to judge the diagnostic accuracy of SNRB. An alternative approach is to determine the sensitivity of SNRB using a 'case' injection at a symptomatic nerve root level where nerve root compression is confirmed by imaging. Specificity is evaluated by a 'control' injection at an asymptomatic site (e.g. adjacent nerve root) where imaging demonstrates no nerve root compression. Hence, in this approach concordant clinical and imaging findings are used as the reference standard.

Two reviewers independently screened titles and abstracts for relevance and full papers for eligibility. Any disagreements were resolved by consensus or referred to the review team.

For peer review only - http://bmjopen.bmj.com/site/about/guidelines.xhtml

Data extraction and quality (bias and applicability) assessment

Data extraction was performed by one reviewer and checked by a second: disagreements were resolved by consensus or discussion among co-authors. We extracted data on: study design, inclusion and exclusion criteria, included patients, SNRB details and reference standard details. 'Per patient' data were extracted: if these were unavailable we extracted 'per injection' data.

Studies included in the diagnostic review were assessed for methodological quality using the QUADAS-2 measure of bias and applicability. Bias occurs if the results of a study are distorted by flaws or limitations in its design or conduct (e.g. knowledge of the index test result when interpreting the reference standard). Applicability may be reduced if patient characteristics, or the use or interpretation of the index test in the study differ from those likely to prevail in clinical practice. Reviewers rate concerns regarding applicability and risk of bias as low, high or unclear. At least two reviewers assessed quality using QUADAS-2 and any disagreements were resolved by consensus.[22]

Studies were judged to be of high applicability if: 1) they recruited patients with low back pain and suspected radiculopathy (sciatica) with non-congruent imaging and clinical findings, who might benefit from lumbar decompression surgery; 2) the SNRB included injection of anaesthetic, sometimes in conjunction with a steroid, close to the lumbar nerve root most often under guidance by fluoroscopy or other imaging; 3) the test aimed to identify patients with radiculopathy (sciatica) that was amenable to surgery; and 4) the reference standard was outcome of surgery. We did not carry out formal quality assessment of studies reporting on adverse events.

Data synthesis and analysis

We performed all analyses in Stata v15.1.[23] We calculated sensitivity and specificity of SNRB from each study and plotted these in receiver-operating characteristic (ROC) space. We performed random effects logistic regression to meta-analyse studies grouped by reference standard,[24] using an updated version of the *metandi* package.[25] Data from studies on adverse events were combined in a narrative summary. We reported our findings according to the Preferred Reporting Items for Systematic Reviews and Meta-Analyses (PRISMA) for Diagnostic Test Accuracy (DTA) Studies.[26]

For peer review only - http://bmjopen.bmj.com/site/about/guidelines.xhtml

Patient and Public Involvement

Patients and members of the public were not involved in this review.

For peer review only

For peer review only - http://bmjopen.bmj.com/site/about/guidelines.xhtml

RESULTS

The original searches identified 12,883 titles and abstracts and an additional 5,267 were identified in the update search in 2018. Overall, 61 titles and abstracts were considered potentially relevant and full papers were retrieved and screened. Our original review included 5 studies. We identified one additional relevant study through our updated searches. A total of 6 studies (total 341 patients, sample size range 15 to 100) were therefore included in the review of diagnostic accuracy (Figure 1). Where reported, the mean age of patients was in the mid-forties, the majority were male, and most had had symptoms for at least 3 months. One study excluded patients with a previous history of lumbar surgery,[27] in contrast a substantial minority of patients (up to 48%) had had previous surgery in two of the other studies. Details of the patients included, and the injections delivered in each study are given in Table 1 (Supplementary Table 1).

Four diagnostic cohort studies (one prospective and three retrospective) recruited patients with suspected lumbar radiculopathy in whom some doubt remained due to equivocal or discordant clinical and radiological findings. Schutz et al. and Dooley et al. used intra-operative findings during surgery as the reference standard (Table 2).[28 29] In addition, Dooley et al. used outcome following surgery as a second reference standard.[29] Williams et al. and Sasso et al. used outcome following surgery at 3 and 12 months,[30 31] respectively, as the reference standard.

Two studies had a within-patient case-control study design. In the Yeom et al. study control injections were given at adjacent asymptomatic nerve roots,[27] whereas in the North et al. study other anatomic sites in the lumbar spine were injected (sciatic nerve, facet joint and subcutaneous).[32] All cases were confirmed by concordant clinical and radiological or surgical findings prior to the use of SNRB.

Quality of included studies

All studies were judged at high risk of bias (Table 3). All studies had high risk of bias for the reference standard because post-surgical outcomes were not considered[27 32] or selectively measured[28-31] (e.g. surgery was predominantly performed in patients with positive SNRB findings). The four cohort studies were at high risk of bias for flow and timing because patients were selected to undergo surgery based on the SNRB result, with patients testing positive more likely to receive surgery. It is likely that the patients with negative SNRB results who, despite this, were selected for surgery were a biased subset of those testing negative as

For peer review only - http://bmjopen.bmj.com/site/about/guidelines.xhtml

these are likely to have been the patients in whom the clinicians suspected a false-negative result. The two within patient case-control studies were at high risk of bias and poor applicability for patient selection because they recruited patients with unequivocal and concordant imaging and clinical findings rather than patients where diagnostic uncertainty remained. Three cohort studies were judged as low concerns regarding applicability on all domains.[29-31] There were high concerns regarding the applicability of the fourth cohort study as the reference standard consisted of intra-operative findings alone.[28]

Summary of test accuracy results

The diagnostic cohort studies reported data at the patient level, but only data at the injection level were available for the within-patient case-control studies. The threshold used to determine a positive SNRB test varied between studies (Table 2). We decided not to pool the results of studies that used different reference standards.

There was substantial heterogeneity in estimates of sensitivity and specificity across studies; sensitivity ranged from 57% to 100% and specificity from 10% to 86% (Table 2, Figure 2). Sensitivity exceeded 85% in all studies except Yeom et al. (57%).[27] Specificity was lower than 75% in all studies except Yeom et al. (86%).[27] Interpretation of specificity is particularly hampered by verification bias in the cohort studies. Because surgeons were not blinded to the SNRB results, very few patients with negative test findings had surgery. Williams et al., Sasso et al., Schutz et al., and Dooley et al. contributed a total of just ten true negative cases[28-31]. The higher specificity reported by Yeom et al. could be a manifestation of patient selection bias as 'control' injections were performed at a level of the spine where the patients had no symptoms or imaging findings suggestive of pathology.[27] Positive likelihood ratios were generally low (<5), meaning that a positive SNRB result did not greatly increase the post-test probability that the nerve root was the source of the low back and radicular pain.

Due to the patient selection bias inherent in within patient case-control designs we decided that it would be inappropriate statistically to combine their results with those of the diagnostic cohort studies, and because of differences in the type of control injection we did not pool the results of the two studies. Based on the two cohort studies that used an intra-operative reference standard the pooled sensitivity was 93.5% (95% CI 84.0% - 97.6%) and specificity was 50.0% (16.8% - 83.2%). For the three studies that used post-surgery as the reference standard

For peer review only - http://bmjopen.bmj.com/site/about/guidelines.xhtml

the summary sensitivity was 90.9% (83.1% - 95.3%) and summary specificity was 22.0% (7.4% - 49.9%). Low specificity implies that a high proportion of patients without nerve root compromise have a positive SNRB result.

For peer review only

For peer review only - http://bmjopen.bmj.com/site/about/guidelines.xhtml

Table 1 Details of included studies

Author (Year) Country	N analysed/ N recruited	Description of included patients	Details of previous surgery	Needle level	Anaesthetic details	Guided method	Needle provocation	Number of control injections	Time to pain measurement
Within patient case-control studies									
Yeom[27] (2008) NR	47/83	Established single-level radiculopathy. Concordant imaging & clinical findings.	No history of lumbar surgeries.	L3, L4, L5, S1	1ml of 2% Lidocaine	Fluoroscopy	No	1 or 2	30 min
North[32] (1996) USA	33/33	Established sciatica with or without low back pain. History of nerve root compression or imaging findings of ongoing nerve root compression.	48% history of root compression corrected surgically.	L5, S1	3ml of 0.5% Bupivacaine	Fluoroscopy	Yes	3	Every 15 min for 3 hours
Prospective diagnostic cohort studies									
Schutz[31] (1973) Canada	15/23	Current sciatica symptoms.	Unclear if patients included in analysis had previous surgeries.	NR	1ml of Procaine (concentration NR)	Guided but method NR	Yes	1 or 2	Immediate
Retrospective diagnostic cohort studies									
Sasso[29] (2005) USA	83/83	Cervical or lumbar radiculopathy. Discordant imaging & clinical findings.	Unclear how many previous lumbar surgeries.	NR	0.5-0.7ml of 2% Lidocaine	Fluoroscopy	Yes	NR	Immediate
Dooley[32] (1988) Canada	62/73	Radicular pain & previous nerve root infiltration.	32 >=1 previous surgery, 3 had 4 surgeries.	L3, L4, L5, S1	1ml of 1% Mepivacaine or Lidocaine	Fluoroscopy	Yes	NR	Immediate
Williams[28] (2015) UK	96/100	Presumed radicular leg pain. Discordant clinical & imaging findings.	NR	L1, L3, L4, L5, S1	2 mL of 1% Lidocaine and 0.5 to 1 mL of Iopamidol	Fluoroscopy	Yes	NR	Immediate

Abbreviations: DRGB, dorsal root ganglion block; NR, not reported; MRI, Magnetic resonance imaging; SNRB, selective nerve root block.

For peer review only - http://bmjopen.bmj.com/site/about/guidelines.xhtml

Table 2 Diagnostic accuracy results

Author (year)	Threshold	Reference standard	TP	FN	Sensitivity % (95% CI)	TN	FP	Specificity % (95% CI)	PLR (95% CI)	NLR (95% CI)
Within-patient case-control studies										
Yeom[27] (2008)	70% pain relief – several other thresholds also evaluated.	Concordant symptoms and imaging evidence of compression (case injections) or no symptoms or imaging evidence of compression (control injections).	27	20	57 (43, 70)	50	8	86 (75, 93)	4.1 (2.1, 8.3)	0.5 (0.4, 0.7)
North[32] (1996)	50% reduction in baseline pain following block.	Concordant symptoms and imaging evidence of compression (case injections) or no symptoms or imaging evidence of compression (control injections).	30	3	91 (76, 97)	8	25	24 (12, 41)	1.2 (1.0, 1.5)	0.4 (0.1, 1.3)
Diagnostic cohort studies										
Schutz[31] (1973)	100% pain relief. Full trunk flexion and straight leg raising possible.	Intraoperative findings.	12	0	100 (76, 100)	1	2	33 (6, 79)	1.5 (0.7, 3.3)	0.0
Sasso[29] (2005)	Visual Analog Scale score 0-1 & immediate relief of >95% pain	Outcome 12 months following surgery	71	3	96 (89, 99)	5	4	56 (27, 81)	2.2 (1.0, 4.5)	0.1 (0.0, 0.3)
Dooley[32] (1988)	Pain relief	Intraoperative surgical confirmation of root pathology	46	4	92 (81, 98)	2	1	67 (9, 99)	2.8 (0.6, 13.7)	0.1 (0.0, 0.4)
		Outcome following surgery (follow up range 24-36 months)	28	4	88 (71, 96)	2	19	10 (1, 30)	1.0 (0.8, 1.2)	1.2 (0.3, 6.5)
Williams[28] (2015)	Pain relief	Outcome 3 months following surgery (resolution of symptoms)	41	7	85 (72, 94)	2	10	17 (3, 48)	1.0 (0.8, 1.4)	0.9 (0.2, 3.7)

*Please note that the unit used in the within patient case-control studies is number of injections and some patients had two control injections at adjacent levels in addition to the affected nerve; in all other studies it is number of patients.
Abbreviations: TP, true positive; FN, false negative; TN, true negative; FP, false positive; CI, confidence interval; PLR, positive likelihood ratio; NLR, negative likelihood ratio.

For peer review only - http://bmjopen.bmj.com/site/about/guidelines.xhtml

Table 3 QUADAS-2 results

Author (year)	RISK OF BIAS				APPLICABILITY CONCERNS		
	Patient selection	Index test	Reference standard	Flow and timing	Patient selection	Index test	Reference standard
Within patient case control studies							
Yeom[27] (2008)	☹	☹	☹	☹	☹	☺	☹
North[31] (1996)	☹	☺	☹	☺	☹	☺	☹
Diagnostic cohort studies							
Sasso[29] (2005)	☺	☺	☹	☹	☺	☺	☺
Schutz[31] (1973)	?	☺	☹	☹	?	☺	☹
Dooley[32] (1988)	☺	☺	☹	☹	☺	☺	☺
Williams[28] (2015)	☺	?	☹	☹	☺	☺	☺

☺ **Low risk/ concern;** ☹ **High risk/ concern; ? Unclear risk/concern**

Adverse events review

Eight studies assessed complications and/or adverse events (Supplementary Table 2).[28 30 33-38] Two were diagnostic cohorts,[28 30] one was a randomized controlled trial[34] and five were case series.[33 35-38] Only one reported the complications of SNRBs in the lumbar spine as the primary outcome.[33] Five studies reported that there were no complications. Tajima et al. reported aggravated pain in the lower extremity for 1-2 days following selective radiculography and block in 4 (3.8%) patients.[38] The largest study reported that minor and transient complications were encountered in 98 of the 1777 total patient visits (during which 2217 injections were delivered to 1203 patients), giving an overall per patient visit complication rate of 5.5%.[33] Complications occurred in 134 of the 2217 total injections (6% complication rate per injection). There were no major or permanent complications resulting from SNRB in this large case series.

For peer review only - http://bmjopen.bmj.com/site/about/guidelines.xhtml

DISCUSSION

Despite the longstanding use of SNRB to help in the selection of patients who might benefit from surgery and in guiding the surgical approach, few studies have estimated its diagnostic accuracy. Our systematic review identified six studies, all at high risk of bias. Many were at risk of verification bias, because patients with positive SNRB were more likely to undergo surgery than those testing negative. There was substantial variation in estimates of sensitivity and specificity across studies. Based on the three cohort studies that used post-surgery outcomes as the reference standard, the summary sensitivity was 90.9% (83.1% - 95.3%) and summary specificity was 22.0% (7.4% - 49.9%). SNRB is a safe test with a low risk of clinically significant complications, but it remains unclear whether the additional diagnostic information it provides, improves patient outcomes or justifies the cost of the test.

Extensive literature searches were conducted in an attempt to locate all relevant studies. These included electronic searches in a wide variety of databases, scanning the references of included studies and previous systematic reviews. Diagnostic accuracy studies are difficult to identify from electronic databases as there are no specific indexing terms. Therefore, very sensitive searches were carried out to ensure that relevant studies were not missed. It is unlikely that any relevant published studies have been missed, although it is possible that some unpublished studies were not identified. The small number of primary diagnostic accuracy studies included in our review, all had methodological limitations. Due to the small number of studies, we were unable to explore the value of SNRB in potentially important patient subgroups, such as those with suspected multi-level radiculopathy.

Four previous systematic reviews of the diagnostic utility of SNRB in patients whose pain was of spinal origin have been reported.[15-18] The two earlier reviews had positive interpretations of the data and concluded that there was moderate evidence for SNRB in the "pre-operative evaluation of patients with negative or inconclusive imaging studies, but with clinical findings of nerve root irritation".[16 18] More recent reviews, however, concluded that there was limited evidence for the accuracy of SNRB as a diagnostic tool.[15 17] Our update review shows similar results. We found limited evidence which was of low methodological quality indicating that the diagnostic accuracy of SNRB is uncertain and that specificity in particular may be low. The differences in interpretation between our review and those conducted previously may be partly due to the smaller number of primary studies included in our review. We used rigorous eligibility criteria, which excluded studies with mixed cervical and

For peer review only - http://bmjopen.bmj.com/site/about/guidelines.xhtml

lumbar spine pathology and studies where there was insufficient data to construct estimates of sensitivity and specificity.

For centres that currently rely on SNRB for diagnostic information to help decide whether, or at which level, to perform lumbar decompressive surgery, it is vital that better evidence is generated. Moreover, according to Hospital Episodes Statistics (HES), which contains records of all admissions, appointments and attendances for patients at NHS hospitals in England, 58,399 injections of therapeutic substance around spinal nerve root took place from 01 April 2016 to 31 March 2017.[8] Due to the granularity of HES data, it is not possible to tell how many of these injections were diagnostic lumbar SNRB. Nevertheless, the number is substantial, and it is therefore apparent that the community of spinal surgeons has a responsibility to generate robust evidence for the use of diagnostic SNRBs. A methodologically ideal diagnostic accuracy study is unlikely to be clinically acceptable as it would require all patients, including those with negative SNRB findings, to undergo surgery. Furthermore, while diagnostic accuracy studies can explore whether SNRB accurately predicts surgical outcomes, they cannot answer the more fundamental question of whether SNRB improves surgical decisions and patient outcomes. Much better evidence would be provided by a trial randomising patients who are being considered for surgery but have discordant or equivocal clinical and imaging findings of nerve root compression to receive a diagnostic SNRB or to have management based on clinical and imaging findings alone. Given the lack of high quality evidence on the diagnostic accuracy of SNRB, we believe that such a trial would be ethically acceptable and would help patients, clinicians and health care payers decide whether SNRB can improve patient outcomes by targeting surgery at those most likely to benefit.

Finally, it should be mentioned that this systematic review did not consider the use of SNRBs as a therapeutic option for patients with radicular pain due to a prolapsed lumbar intervertebral disc. The most recent NICE guidance concluded that the evidence for both image guided and non-image guided injections for patients with acute and severe sciatica was mostly low or moderate.[8] However, the guidance recommends that an injection of local anaesthetic and steroid should be considered in acute, severe sciatica where patients would otherwise be offered surgery. The NERVES randomised trial, which enrolled patients in 12 NHS hospitals, aimed to compare surgical microdiscectomy versus SNRB in patients with sciatica of at least 6 weeks' duration secondary to a prolapsed intervertebral disc. The results of this trial, which is currently in follow-up, will elucidate the role of

For peer review only - http://bmjopen.bmj.com/site/about/guidelines.xhtml

SNRB as a therapeutic but not diagnostic option. Hence, it is important that consideration is given to a trial of diagnostic SNRB as outlined above.

CONCLUSIONS

There is no high-quality evidence on the diagnostic accuracy of SNRB in patients with radiculopathy and discordant or equivocal imaging findings. The evidence that is available suggests that the specificity of SNRB is low. As there is no adequate reference standard for determining the diagnostic accuracy of SNRB, future research should focus on randomised controlled trials to evaluate whether SNRB improves the process of care or patient outcomes.

For peer review only

For peer review only - http://bmjopen.bmj.com/site/about/guidelines.xhtml

Funding

PW and ME are funded by a National Institute for Health Research Collaboration for Leadership in Applied Health Research and Care West (NIHR CLAHRC West). AS is funded by an NIHR Systematic Review Fellowship (RM-SR-2017-08-012). AK is supported by a Clinical Lectureship, School of Clinical Medicine, University of Cambridge. NH, RB, RL, PW and WH were funded by a National Institute for Health Research Health Technology Assessment programme grant (project number 09/111/01).

Competing interests

None declared

Data availability statement

There is no identifiable patient data included in the manuscript. All included data are extracted from published trials and references are included.

Author contributions

RB conducted the reviews of diagnostic accuracy and adverse events, conducted analyses and completed the first draft of the manuscript. ME updated the review, including abstract and full text screening, data extraction, risk of bias assessment, analysis and updated the draft of the manuscript. AS updated the review, including abstract and full text screening and checking of data extraction and risk of bias assessment. RL contributed to the conception and design of the study, provided clinical expertise in neurosurgery, helped with acquisition of data for the service evaluation of SNRB and critically revised the manuscript. AK provided clinical expertise in neurosurgery and critically revised the manuscript. NH was involved in the inception of study and critical appraisal of literature, particularly from a radiological perspective. PW contributed to the conception and design of the study, supervised conduct of reviews of diagnostic accuracy and adverse events, and critically revised the manuscript. WH was principal investigator on the project, contributed to the conception and design of the study, supervised conduct of reviews of diagnostic accuracy and adverse events and critically revised the manuscript. All authors approved the manuscript for publication.

For peer review only

For peer review only - http://bmjopen.bmj.com/site/about/guidelines.xhtml

Acknowledgements

The authors wish to thank Margaret Burke for her advice in developing and implementing the search Strategy, Catherine Jameson for implementing the original searches and reviews and Alison Richards for conducting the update searches. This research was supported by the National Institute for Health Research (NIHR) Collaboration for Leadership in Applied Health Research and Care West (NIHR CLAHRC West) and the NIHR Health Technology Assessment programme. The views expressed in this article are those of the author(s) and not necessarily those of the NHS, the NIHR, or the Department of Health and Social Care.

For peer review only

For peer review only - http://bmjopen.bmj.com/site/about/guidelines.xhtml

REFERENCES

1. Maniadakis N, Gray A. The economic burden of back pain in the UK. *Pain* 2000;84(1):95-103. doi: S0304-3959(99)00187-6 [pii] [published Online First: 1999/12/22]
2. Jacobs WC, van Tulder M, Arts M, et al. Surgery versus conservative management of sciatica due to a lumbar herniated disc: a systematic review. *Eur Spine J* 2011;20(4):513-22. doi: 10.1007/s00586-010-1603-7 [published Online First: 2010/10/16]
3. Weinstein JN, Tosteson TD, Lurie JD, et al. Surgical versus nonsurgical therapy for lumbar spinal stenosis. *N Engl J Med* 2008;358(8):794-810. doi: 10.1056/NEJMoa0707136 [published Online First: 2008/02/22]
4. Weinstein JN, Tosteson TD, Lurie JD, et al. Surgical vs nonoperative treatment for lumbar disk herniation: the Spine Patient Outcomes Research Trial (SPORT): a randomized trial. *JAMA* 2006;296(20):2441-50. doi: 10.1001/jama.296.20.2441 [published Online First: 2006/11/23]
5. Peul WC, van Houwelingen HC, van den Hout WB, et al. Surgery versus prolonged conservative treatment for sciatica. *N Engl J Med* 2007;356(22):2245-56. doi: 10.1056/NEJMoa064039 [published Online First: 2007/06/01]
6. Peul WC, van den Hout WB, Brand R, et al. Prolonged conservative care versus early surgery in patients with sciatica caused by lumbar disc herniation: two year results of a randomised controlled trial. *British Medical Journal* 2011;336(7657)
7. Atlas SJ, Keller RB, Chang Y, et al. Surgical and nonsurgical management of sciatica secondary to a lumbar disc herniation: five-year outcomes from the Maine Lumbar Spine Study. *Spine (Phila Pa 1976)* 2001;26(10):1179-87. [published Online First: 2001/06/20]
8. The National Institute for Health and Care Excellence (NICE). Low back pain and sciatica in over 16s: assessment and management (NG59). 2016
9. Parker SL, Mendenhall SK, Godil SS, et al. Incidence of Low Back Pain After Lumbar Discectomy for Herniated Disc and Its Effect on Patient-reported Outcomes. *Clin Orthop Relat Res* 2015;473(6):1988-99. doi: 10.1007/s11999-015-4193-1 [published Online First: 2015/02/20]
10. Baber Z, Erdek MA. Failed back surgery syndrome: current perspectives. *J Pain Res* 2016;9:979-87. doi: 10.2147/JPR.S92776 [published Online First: 2016/11/18]
11. Deyo RA, Diehl AK. Lumbar Spine Films in Primary Care - Current Use and Effects of Selective Ordering Criteria. *Journal of General Internal Medicine* 1986;1(1):20-25. doi: 10.1007/Bf02596320
12. Vroomen PC, de Krom MC, Wilmink JT, et al. Diagnostic value of history and physical examination in patients suspected of lumbosacral nerve root compression. *J Neurol Neurosurg Psychiatry* 2002;72(5):630-4. [published Online First: 2002/04/24]
13. Rubinstein SM, van Tulder M. A best-evidence review of diagnostic procedures for neck and low-back pain. *Best Pract Res Clin Rheumatol* 2008;22(3):471-82. doi: 10.1016/j.berh.2007.12.003 [published Online First: 2008/06/04]
14. Boswell MV, Trescot AM, Datta S, et al. Interventional techniques: evidence-based practice guidelines in the management of chronic spinal pain. *Pain Physician* 2007;10(1):7-111. [published Online First: 2007/01/27]
15. Beynon R, Hawkins J, Laing R, et al. The diagnostic utility and cost-effectiveness of selective nerve root blocks in patients considered for lumbar decompression surgery: a systematic review and economic model. *Health Technol Assess* 2013;17(19):1-88, v-vi. doi: 10.3310/hta17190 [published Online First: 2013/05/16]
16. Datta S, Everett CR, Trescot AM, et al. An updated systematic review of the diagnostic utility of selective nerve root blocks. *Pain Physician* 2007;10(1):113-28. [published Online First: 2007/01/27]
17. Datta S, Manchikanti L, Falco FJ, et al. Diagnostic utility of selective nerve root blocks in the diagnosis of lumbosacral radicular pain: systematic review and update of current evidence. *Pain Physician* 2013;16(2 Suppl):SE97-124. [published Online First: 2013/05/03]
18. Everett CR, Shah RV, Sehgal N, et al. A systematic review of diagnostic utility of selective nerve root blocks. *Pain Physician* 2005;8(2):225-33. [published Online First: 2006/07/20]
19. Leeflang MMG, Scholten RJPM, Rutjes AWS, et al. Use of methodological search filters to identify diagnostic accuracy studies can lead to the omission of relevant studies. *J Clin Epidemiol* 2006;59(3):234-40. doi: 10.1016/j.jclinepi.2005.07.014

For peer review only

For peer review only - http://bmjopen.bmj.com/site/about/guidelines.xhtml

20. Whiting P, Westwood M, Beynon R, et al. Inclusion of methodological filters in searches for diagnostic test accuracy studies misses relevant studies. *J Clin Epidemiol* 2011;64(6):602-7. doi: 10.1016/j.jclinepi.2010.07.006 [published Online First: 2010/11/16]
21. Beynon R, Leeflang MM, McDonald S, et al. Search strategies to identify diagnostic accuracy studies in MEDLINE and EMBASE. *Cochrane Database Syst Rev* 2013(9):MR000022. doi: 10.1002/14651858.MR000022.pub3 [published Online First: 2013/09/12]
22. Whiting PF, Rutjes AW, Westwood ME, et al. QUADAS-2: a revised tool for the quality assessment of diagnostic accuracy studies. *Ann Intern Med* 2011;155(8):529-36. doi: 10.7326/0003-4819-155-8-201110180-00009 [published Online First: 2011/10/19]
23. StataCorp. Stata Statistical Software: Release 15. *College Station, TX: StataCorp LLC* 2017
24. DerSimonian R, Laird N. Meta-analysis in clinical trials. *Control Clin Trials* 1986;7(3):177-88. [published Online First: 1986/09/01]
25. Harbord RM, Whiting P. metandi: Meta-analysis of diagnostic accuracy using hierarchical logistic regression. *Stata Journal* 2009;9(2):211-29.
26. McInnes MDF, Moher D, Thombs BD, et al. Preferred Reporting Items for a Systematic Review and Meta-analysis of Diagnostic Test Accuracy Studies: The PRISMA-DTA Statement. *JAMA* 2018;319(4):388-96. doi: 10.1001/jama.2017.19163 [published Online First: 2018/01/25]
27. Yeom JS, Lee JW, Park KW, et al. Value of diagnostic lumbar selective nerve root block: a prospective controlled study. *AJNR Am J Neuroradiol* 2008;29(5):1017-23. doi: 10.3174/ajnr.A0955 [published Online First: 2008/02/15]
28. Schutz H, Lougheed WM, Wortzman G, et al. Intervertebral nerve-root in the investigation of chronic lumbar disc disease. *Can J Surg* 1973;16(3):217-21. [published Online First: 1973/05/01]
29. Dooley JF, McBroom RJ, Taguchi T, et al. Nerve root infiltration in the diagnosis of radicular pain. *Spine (Phila Pa 1976)* 1988;13(1):79-83. [published Online First: 1988/01/01]
30. Williams AP, Germon T. The value of lumbar dorsal root ganglion blocks in predicting the response to decompressive surgery in patients with diagnostic doubt. *Spine J* 2015;15(3 Suppl):S44-9. doi: 10.1016/j.spinee.2015.01.004 [published Online First: 2015/01/13]
31. Sasso RC, Maradaeg K, Nordmann D, et al. Selective nerve root injections can predict surgical outcome for lumbar and cervical radiculopathy - Comparison to magnetic resonance imaging. *Journal of Spinal Disorders & Techniques* 2005;18(6):471-78. doi: 10.1097/01.bsd.0000146761.36658.45
32. North RB, Kidd DH, Zahurak M, et al. Specificity of diagnostic nerve blocks: A prospective, randomized study of sciatica due to lumbosacral spine disease. *Pain* 1996;65(1):77-85. doi: 10.1016/0304-3959(95)00170-0
33. Stalcup ST, Crall TS, Gilula L, et al. Influence of needle-tip position on the incidence of immediate complications in 2,217 selective lumbar nerve root blocks. *Spine J* 2006;6(2):170-6. doi: 10.1016/j.spinee.2005.08.009 [published Online First: 2006/03/07]
34. Ghahreman A, Ferch R, Bogduk N. The efficacy of transforaminal injection of steroids for the treatment of lumbar radicular pain. *Pain Med* 2010;11(8):1149-68. doi: 10.1111/j.1526-4637.2010.00908.x [published Online First: 2010/08/14]
35. Jonsson B, Stromqvist B, Annertz M, et al. Diagnostic lumbar nerve root block. *J Spinal Disord* 1988;1(3):232-5. [published Online First: 1988/01/01]
36. Ng LC, Sell P. Outcomes of a prospective cohort study on peri-radicular infiltration for radicular pain in patients with lumbar disc herniation and spinal stenosis. *Eur Spine J* 2004;13(4):325-9. doi: 10.1007/s00586-003-0649-1 [published Online First: 2004/01/10]
37. Quinn SF, Murtagh FR, Chatfield R, et al. CT-guided nerve root block and ablation. *AJR Am J Roentgenol* 1988;151(6):1213-6. doi: 10.2214/ajr.151.6.1213 [published Online First: 1988/12/01]
38. Tajima T, Furukawa K, Kuramochi E. Selective lumbosacral radiculography and block. *Spine (Phila Pa 1976)* 1980;5(1):68-77. [published Online First: 1980/01/01]

For peer review only

For peer review only - http://bmjopen.bmj.com/site/about/guidelines.xhtml

Figure legends

Figure 1 Flowchart of diagnostic study selection process

Figure 2 ROC plot displaying diagnostic accuracy results of included studies. Abbreviations: PS, Post-surgical reference standard; IO, Intraoperative reference standard; CI, Control injection reference standard.

For peer review only

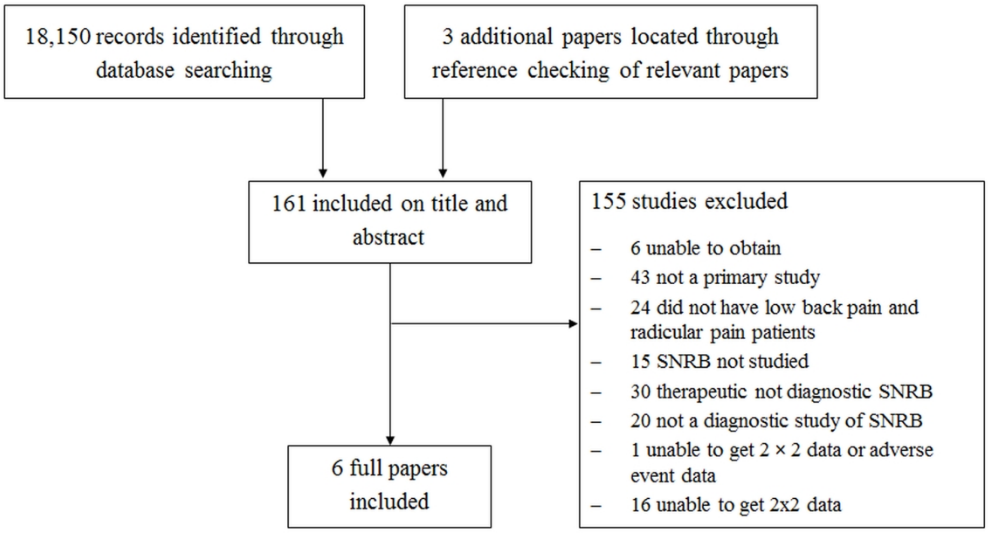

Flowchart of diagnostic study selection process

165x90mm (300 x 300 DPI)

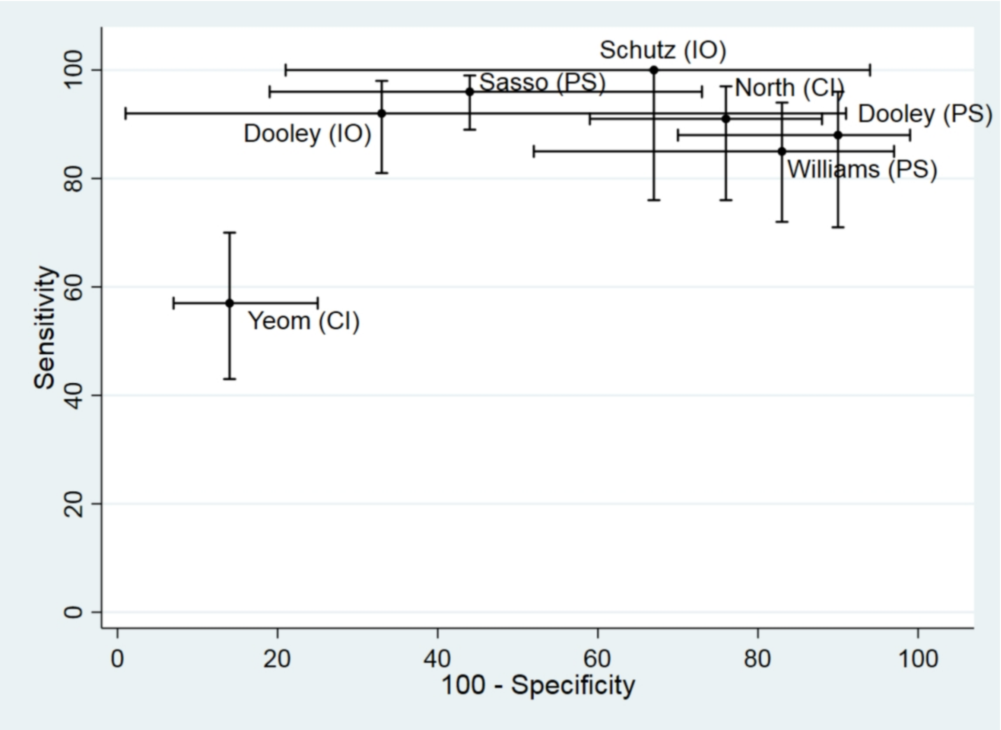

ROC plot displaying diagnostic accuracy results of included studies. Abbreviations: PS, Post-surgical reference standard; IO, Intraoperative reference standard; CI, Control injection reference standard.

123x90mm (300 x 300 DPI)

Supplementary Table 1 Details of included studies

Author (Year) Country	N analysed/N recruited	Inclusion criteria	Description of included patients	Details of previous surgery	Needle level	Anaesthetic details	Guided method	Needle provocation	Number of control injections	Time to pain measurement
Within patient case-control studies										
Yeom[26] (2008) NR	47/83	Patients due to undergo a lumbar spine operation with single-level, unilateral lumbosacral radiculopathy confirmed by clinical, radiographic & MRI findings.	Patients with established pure radiculopathy from a single level. Affected roots were L4 in 3, L5 in 31, S1 in 13. Concordant imaging & clinical findings.	No history of lumbar surgeries	L3, L4, L5, S1	1ml of 2% Lidocaine	Fluoroscopy	No	1 or 2	30 minutes
North[29] (1996) USA	33/33	Patients with sciatica with or without low back pain, attributed to spinal pathology.	Established sciatica patients with or without low back pain. All had L5 or S1 radiculopathy. 52% had diagnostic imaging findings of ongoing nerve root compression. The remaining 48% had a well-documented history of root compression which had been corrected surgically.	48% history of root compression corrected surgically.	L5, S1	3ml of 0.5% Bupivacaine	Fluoroscopy	Yes	3	Every 15 minutes for 3 hours
Prospective diagnostic cohort studies										
Schutz[31] (1973) Canada	15/23	Patients with current sciatica.	Patients with sciatica. Investigation undertaken only at a time when sciatica symptoms actually present.	1 patient had previous surgery, unsuccessful SNRB & excluded from analysis. Unclear if patients	NR	1ml of Procaine (concentrati	Guided but method not reported	Yes	1 or 2	Immediate

Author (Year) Country	N analysed/N recruited	Inclusion criteria	Description of included patients	Details of previous surgery	Needle level	Anaesthetic details	Guided method	Needle provocation	Number of control injections	Time to pain measurement
				included in analysis had previous surgeries.		on not reported)				
Retrospective diagnostic cohort studies										
Sasso[29] (2005) USA	83/83	Patients who underwent SNRB, MRI & nerve root decompression surgery and had a follow-up evaluation >12 months post surgery	Patients with cervical or lumbar radiculopathy. Discordant imaging and clinical findings	Unclear how many previous lumbar surgeries. 20 patients with cervical or lumbar symptoms had previous surgery	NR	0.5-0.7ml of 2% Lidocaine	Fluoroscopy	Yes	NR	Immediate
Dooley[32] (1988) Canada	62/73	Patients who underwent nerve root infiltration	Patients with radicular pain who underwent nerve root infiltration	32 >=1 previous surgery, 3 had 4 surgeries.	L3, L4, L5, S1	1ml of 1% Mepivacaine or Lidocaine	Fluoroscopy	Yes	NR	Immediate
Williams[28] (2015) UK	96/100	Patients who underwent diagnostic lumbar DRGB (identified retrospectively)	Patients with presumed radicular leg pain with significant diagnostic uncertainty from the patient's presenting history, examination and imaging as to whether lumbosacral nerve root compression was indeed responsible.	NR	L1, L3, L4, L5, S1	2 mL of 1% Lidocaine and 0.5 to 1 mL of Iopamidol	Fluoroscopy	Yes	NR	Immediate

Abbreviations: DRGB, dorsal root ganglion block; NR, not reported; MRI, Magnetic resonance imaging; SNRB, selective nerve root block.

For peer review only

For peer review only - http://bmjopen.bmj.com/site/about/guidelines.xhtml

Supplementary Table 2 Patient characteristics of studies reporting on adverse events following SNRB

Author (year) Country	N analysed/ N recruited	Inclusion criteria	Needle tip position	Needle levels	Anaesthetic details	Guided method	Needle provocation used?	Adverse events
Case-series								
Stalcup[32] (2006) USA	1203/ 1203	All adult patients who underwent a SNRB in the lumbar spine in a radiology department.	Adjacent or into the intervertebral foramen.	NR	1-2ml of 0.25% Bupivacaine	Fluoroscopy	NR	Numbers given in injections: Leg weakness n=77; Pain increased n=51; Other n=6; Total n=134
Ng[35] (2004) UK	117/ 125	Consecutive patients with clinical evidence of unilateral radicular pain that lasted despite at least 6 weeks of conservative management, MRI confirmation of nerve root compression secondary to lumbar disc herniation or peripheral degenerative spinal stenosis.	Superiorly to pedicle, medially to nerve and laterally to vertebral body.	NR	2ml of 0.25% Bupivacaine	Assumed Fluoroscopy	NR	No adverse events
Jonsson[34] (1988) Sweden	78/ 78	Patients with unilateral sciatic pain, considered for surgery. Sciatic pain but normal findings on myelography, CT and/or MRI.	Just lateral to the opening of the intervertebral foramen.	L4, L5, S1	3-6ml of Carbocaine (% NR)	Fluoroscopy	NR	No adverse events.
Quinn[36] (1988) USA	33/ 33	Patients with a herniated disc or foraminal stenosis (n=2) as identified by CT or MRI.	An attempt was made to pierce the nerve or to have the needle tip within 1-2mm of the nerve.	NR	2.5-5ml of 1% Lidocaine or 0.5% Bupivacaine	CT	Yes	No adverse events.
Tajima[37] (1980) Japan	106/ 106	Patients with radicular symptoms undergoing lumbosacral radiculography and block who had lumbosacral diseases.	Approx 4cm lateral to upper margin of lumbar spinous process corresponding to nerve root to be radiographed.	L4, L5, S1	3ml of 1% Lidocaine	x-ray	Yes	Pain in the lower extremity was aggravated for 1-2 days

For peer review only

								following selective radiculography and block in 4 patients. There was no other complication.
Diagnostic cohort study								
*Schutz[31] (1973) Canada	15/ 23	Patients with current sciatica.	Superior level of intervertebral foramen. Introduced about 2” from the midline.	NR	1ml Procaine	Guided but method not reported	Yes	No adverse events.
*Williams[28] (2015) UK	96/100	Patients with presumed radicular leg pain who underwent diagnostic lumbar DRGB (identified retrospectively).	Inserted from a paraspinal entry point and advanced to the superoanterior margin of the intervertebral foramen of the targeted level.	L1,L3, L4,L5 & S1	2 mL of 1% Lidocaine and 0.5 to 1 mL of Iopamidol	Fluoroscopy	Yes	No adverse events.
Randomized controlled trial								
Ghahreman[33] (2010) Australia	27/ 150	Adult patients with lower limb radiculopathy; limitation of straight-leg raise to <30º; disc herniation on CT or MRI. Considered for surgery. Only data for single arm of trial in which patients received anaesthetic was included in the current review.	Placed in the intervertebral foramen of the target level.	L2,L3, L4,L5 & S1	2ml of 0.5% Bupivacaine	Assumed Fluoroscopy	NR	No complications occurred that could be attributed to the treatment.

*Included in diagnostic accuracy systematic review
Abbreviations: CT, computerised tomography; DRGB, dorsal root ganglion block; MRI, magnetic resonance imaging; NR, not reported; SNRB, selective nerve root block.

For peer review only

ELECTRONIC DATABASE SEARCH STRATEGY:
Spinal nerve block- diagnostic utility in back pain

Database: Ovid MEDLINE(R) Epub Ahead of Print, In-Process & Other Non-Indexed Citations, Ovid MEDLINE(R) Daily and Ovid MEDLINE(R) <1946 to Present>

Search Strategy:

----

1 exp Back Pain/ (34777)
2 back pain.tw. (39654)
3 backache.tw. (2333)
4 Radiculopathy/ (4527)
5 Lumbar Vertebrae/ (46628)
6 Lumbosacral Region/ (11245)
7 radiculopath$.tw. (5409)
8 lumbago.tw. (1264)
9 lumber.tw. (1065)
10 lumbosacral.tw. (10151)
11 radiculitis.tw. (781)
12 (radicular adj3 pain).tw. (2652)
13 spinal pain.tw. (1247)
14 exp Spinal Nerve Roots/ (29515)
15 Sciatica/ (4816)
16 lumbar.tw. (93988)
17 sciatica.tw. (3914)
18 Intervertebral Disk Displacement/ (17468)
19 Zygapophyseal Joint/ (1525)
20 Spinal Stenosis/ (5278)
21 Foraminal Stenosis.tw. (515)
22 Foramenal Stenosis.tw. (3)
23 lateral recess stenosis.tw. (124)
24 or/1-23 (195289)
25 exp Nerve Block/ (19509)
26 (nerve adj3 block$).tw. (11732)
27 SNRB.tw. (39)
28 (transforaminal adj3 injection$).tw. (523)
29 Injections, Epidural/ (2600)
30 (neural adj3 block$).tw. (979)
31 (nerve adj3 injection$).tw. (1375)
32 (nerve adj3 infiltration).tw. (583)
33 (block adj3 an?esthetic$).tw. (1128)

For peer review only

34 exp Injections, Spinal/ (15080)
35 facet block$.tw. (80)
36 facet injection$.tw. (69)
37 epidural injection$.tw. (1674)
38 Injections, Intra-Articular/ (6865)
39 diagnostic injection$.tw. (139)
40 or/25-39 (49688)
41 24 and 40 (6586)
42 exp Anesthetics, Local/ (99162)
43 lidocaine.tw. (19794)
44 lignocaine.tw. (2740)
45 local an?esthetic$.tw. (22917)
46 bupivacaine.tw. (11894)
47 exp Triamcinolone/ (8898)
48 Triamcinolone.tw. (6896)
49 volon.tw. (33)
50 aristocort.tw. (22)
51 Depo-medrone.tw. (16)
52 Depomedrone.tw. (20)
53 Steroids/ (34837)
54 Depo steroid$.tw. (7)
55 Deposteroid$.tw. (8)
56 kenalog.tw. (195)
57 kenacort.tw. (59)
58 Depo-Medrol.tw. (146)
59 depomedrol.tw. (40)
60 exp Betamethasone/ (6913)
61 betamethasone.tw. (4443)
62 exp prednisolone/ (48285)
63 prednisolone.tw. (23608)
64 methylprednisolone.tw. (14255)
65 ((steroid$ or corticosteroid$) adj5 (injection$ or infiltration or block)).tw. (8468)
66 or/42-65 (233944)
67 Diagnosis/ (17032)
68 diagnosis, differential/ (424334)
69 diagnosis.fs. (2333150)
70 diagnos$.tw. (2126562)
71 or/67-70 (3705304)
72 66 and 71 (36366)
73 24 and 72 (1633)

For peer review only

74 41 or 73 (7546)

75 exp animals/ not humans/ (4436130)

76 74 not 75 (6125)

77 (20101* or 2011* or 2012* or 2013* or 2014* or 2015* or 2016* or 2017* or 2018*).ep,ez,dc,dp. (7686055)

78 76 and 77 (2085)

Proquest Dissertations and Theses Global

all(backache OR lumbar OR "back pain" OR radiculopathy) AND all("transforaminal injection" OR "back pain infiltration" OR "back pain block" OR "facet injection" OR SNRB OR "nerve block" OR "nerve root block" OR "nerve infiltration" OR "selective nerve root infiltration" OR "facet block" OR "radiculopathy block" OR "radiculopathy infiltration")

For peer review only

PRISMA-DTA Checklist

Section/topic	#	PRISMA-DTA Checklist Item	Reported on page #
**TITLE / ABSTRACT**			
Title	1	Identify the report as a systematic review (+/- meta-analysis) of diagnostic test accuracy (DTA) studies.	P1
Abstract	2	Abstract: See PRISMA-DTA for abstracts.	P2
**INTRODUCTION**			
Rationale	3	Describe the rationale for the review in the context of what is already known.	P4
Clinical role of index test	D1	State the scientific and clinical background, including the intended use and clinical role of the index test, and if applicable, the rationale for minimally acceptable test accuracy (or minimum difference in accuracy for comparative design).	P4
Objectives	4	Provide an explicit statement of question(s) being addressed in terms of participants, index test(s), and target condition(s).	P5
**METHODS**			
Protocol and registration	5	Indicate if a review protocol exists, if and where it can be accessed (e.g., Web address), and, if available, provide registration information including registration number.	NA
Eligibility criteria	6	Specify study characteristics (participants, setting, index test(s), reference standard(s), target condition(s), and study design) and report characteristics (e.g., years considered, language, publication status) used as criteria for eligibility, giving rationale.	P6
Information sources	7	Describe all information sources (e.g., databases with dates of coverage, contact with study authors to identify additional studies) in the search and date last searched.	P6
Search	8	Present full search strategies for all electronic databases and other sources searched, including any limits used, such that they could be repeated.	P6
Study selection	9	State the process for selecting studies (i.e., screening, eligibility, included in systematic review, and, if applicable, included in the meta-analysis).	P6
Data collection process	10	Describe method of data extraction from reports (e.g., piloted forms, independently, in duplicate) and any processes for obtaining and confirming data from investigators.	P6
Definitions for data extraction	11	Provide definitions used in data extraction and classifications of target condition(s), index test(s), reference standard(s) and other characteristics (e.g. study design, clinical setting).	P7
Risk of bias and applicability	12	Describe methods used for assessing risk of bias in individual studies and concerns regarding the applicability to the review question.	P7
Diagnostic accuracy measures	13	State the principal diagnostic accuracy measure(s) reported (e.g. sensitivity, specificity) and state the unit of assessment (e.g. per-patient, per-lesion).	P7
Synthesis of results	14	Describe methods of handling data, combining results of studies and describing variability between studies. This could include, but is not limited to: a) handling of multiple definitions of target condition. b) handling of multiple thresholds of test positivity, c) handling multiple index test readers, d) handling of indeterminate test results, e) grouping and comparing tests, f) handling of different reference standards	P7

For peer review only

For peer review only - http://bmjopen.bmj.com/site/about/guidelines.xhtml

PRISMA-DTA Checklist

Section/topic	#	PRISMA-DTA Checklist Item	Reported on page #
Meta-analysis	D2	Report the statistical methods used for meta-analyses, if performed.	P7
Additional analyses	16	Describe methods of additional analyses (e.g., sensitivity or subgroup analyses, meta-regression), if done, indicating which were pre-specified.	P7
**RESULTS**			
Study selection	17	Provide numbers of studies screened, assessed for eligibility, included in the review (and included in meta-analysis, if applicable) with reasons for exclusions at each stage, ideally with a flow diagram.	P8
Study characteristics	18	For each included study provide citations and present key characteristics including: a) participant characteristics (presentation, prior testing), b) clinical setting, c) study design, d) target condition definition, e) index test, f) reference standard, g) sample size, h) funding sources	P8,9,10
Risk of bias and applicability	19	Present evaluation of risk of bias and concerns regarding applicability for each study.	P8,9,12
Results of individual studies	20	For each analysis in each study (e.g. unique combination of index test, reference standard, and positivity threshold) report 2x2 data (TP, FP, FN, TN) with estimates of diagnostic accuracy and confidence intervals, ideally with a forest or receiver operator characteristic (ROC) plot.	P11
Synthesis of results	21	Describe test accuracy, including variability; if meta-analysis was done, include results and confidence intervals.	P9
Additional analysis	23	Give results of additional analyses, if done (e.g., sensitivity or subgroup analyses, meta-regression; analysis of index test: failure rates, proportion of inconclusive results, adverse events).	P9,12
**DISCUSSION**			
Summary of evidence	24	Summarize the main findings including the strength of evidence.	P13
Limitations	25	Discuss limitations from included studies (e.g. risk of bias and concerns regarding applicability) and from the review process (e.g. incomplete retrieval of identified research).	P13,14
Conclusions	26	Provide a general interpretation of the results in the context of other evidence. Discuss implications for future research and clinical practice (e.g. the intended use and clinical role of the index test).	P14,15
**FUNDING**			
Funding	27	For the systematic review, describe the sources of funding and other support and the role of the funders.	P16

Adapted From: McInnes MDF, Moher D, Thombs BD, McGrath TA, Bossuyt PM, The PRISMA-DTA Group (2018). Preferred Reporting Items for a Systematic Review and Meta-analysis of Diagnostic Test Accuracy Studies: The PRISMA-DTA Statement. JAMA. 2018 Jan 23;319(4):388-396. doi: 10.1001/jama.2017.19163.

For more information, visit: www.prisma-statement.org.

For peer review only - http://bmjopen.bmj.com/site/about/guidelines.xhtml
